# The Malarial Serine Protease SUB1 Plays an Essential Role in Parasite Liver Stage Development

**DOI:** 10.1371/journal.ppat.1003811

**Published:** 2013-12-12

**Authors:** Catherine Suarez, Katrin Volkmann, Ana Rita Gomes, Oliver Billker, Michael J. Blackman

**Affiliations:** 1 Division of Parasitology, Medical Research Council National Institute for Medical Research, Mill Hill, London, United Kingdom; 2 Wellcome Trust Sanger Institute, Hinxton, Cambridge, United Kingdom; Albert Einstein College of Medicine, United States of America

## Abstract

Transmission of the malaria parasite to its vertebrate host involves an obligatory exoerythrocytic stage in which extensive asexual replication of the parasite takes place in infected hepatocytes. The resulting liver schizont undergoes segmentation to produce thousands of daughter merozoites. These are released to initiate the blood stage life cycle, which causes all the pathology associated with the disease. Whilst elements of liver stage merozoite biology are similar to those in the much better-studied blood stage merozoites, little is known of the molecular players involved in liver stage merozoite production. To facilitate the study of liver stage biology we developed a strategy for the rapid production of complex conditional alleles by recombinase mediated engineering in *Escherichia coli*, which we used in combination with existing *Plasmodium berghei* deleter lines expressing Flp recombinase to study subtilisin-like protease 1 (SUB1), a conserved *Plasmodium* serine protease previously implicated in blood stage merozoite maturation and egress. We demonstrate that SUB1 is not required for the early stages of intrahepatic growth, but is essential for complete development of the liver stage schizont and for production of hepatic merozoites. Our results indicate that inhibitors of SUB1 could be used in prophylactic approaches to control or block the clinically silent pre-erythrocytic stage of the malaria parasite life cycle.

## Author Summary

Malaria is caused by a single-celled parasite and is transmitted by the bite of an infected mosquito. The inoculated sporozoite forms of the parasite invade liver cells where they replicate, eventually releasing thousands of merozoites into the bloodstream to initiate the blood stage parasite life cycle which causes clinical malaria. The liver stage of the parasite life cycle is asymptomatic, so it is widely considered a potential target for prophylactic vaccine- or drug-based approaches designed to prevent infection. In this study, we use a robust, highly efficient gene engineering approach called recombineering, combined with a conditional gene deletion strategy to examine the function in liver stages of a parasite protease called SUB1, previously implicated in release of blood stage parasites. We show that SUB1 is expressed in the liver stage schizont and that the protease is essential for production of liver stage merozoites. Our results enhance our understanding of malarial liver stage biology, provide new tools for studying essential gene function in malaria, and suggest that inhibitors of SUB1 could be used as prophylactic drugs to prevent clinical malaria.

## Introduction

Transmission of the malaria parasite to a vertebrate host is initiated by the bite of an infected Anopheline mosquito. The inoculated sporozoites migrate from the site of inoculation, enter the circulation, and are arrested in liver sinusoids where they traverse the vascular endothelium and invade hepatocytes, coming to rest within an intracellular membrane-bound parasitophorous vacuole (PV) [1], [2]. After an initial period of non-replicative development, which lasts around 24 h in the rodent malaria species *Plasmodium berghei*, the intracellular parasite - now known as an exoerythrocytic form (EEF) - initiates an asexual replicative program. This begins with several rounds of nuclear division to form a multinucleated syncytium or schizont, concomitant with a large increase in the size of the PV to accommodate the growing parasite. Approximately 55 h following hepatocyte invasion (in hepatoma cells) the single plasma membrane of the schizont begins to invaginate around groups of parasite nuclei to form the so-called cytomere stage [3], [4]. Subsequent further invagination of the parasite plasma membrane produces clearly defined individual merozoites tightly packed within the PV. Shortly thereafter, the PV membrane (PVM) disintegrates, releasing the merozoites to move freely within the host cell cytoplasm. PVM rupture triggers an unusual form of cell death in the host cell, involving DNA condensation, disintegration of host cell mitochondria and loss of plasma membrane proteins, but lacking certain other classical features of apoptosis such as caspase activation and loss of host plasma membrane phospholipid asymmetry [4], [5], [6]. *In vitro*, infected hepatoma cells such as HepG2 cells round up at this point and detach from their monolayers to float freely in the cultures [4], [5]. Just prior to detachment, merozoite-filled vesicles called merosomes, each surrounded by membrane of host cell origin, are extruded from the host cells. *In vivo*, these enter the lumen of the liver sinusoids from where they are carried to the pulmonary microvasculature to rupture, allowing egress of their merozoite cargo [4], [5], [6]. The merozoites invade erythrocytes to initiate the asexual blood stage cycle. The entire liver stage has a duration of between 2 and 15 days [7], [8], depending on the *Plasmodium* species, and culminates in the production and release of thousands of hepatic merozoites from each infected hepatocyte. Whilst not itself associated with any pathology, the liver stage and other pre-erythrocytic stages are a prerequisite to the asexual blood-stage cycle in a natural malarial infection, and so are potential targets for prophylactic immune-based or chemotherapeutic interventions designed to prevent disease.

Compared to *Plasmodium* asexual blood stages, liver stage malaria parasites are relatively difficult to access [7], [8] and so, despite these elegant and detailed morphological descriptions of the hepatic malaria life cycle, little is known of the signals and molecular players involved in liver stage merozoite development, PVM rupture, merosome formation and merozoite egress. The limited available data suggest that in many respects liver stage merozoites are probably very similar in makeup to their well-studied blood stage counterparts [9]. Elements of merozoite morphogenesis and egress are therefore likely shared between the liver and blood stages. As an example of this, treatment of mature hepatic or erythrocytic schizonts with the cysteine protease inhibitor E64 prevents PVM rupture [5], [10], [11], implicating a common role for cysteine protease(s) in merozoite release. The effects of E64 may result from inhibition of host cell calpain-1 activity, which has been implicated in egress [12], as well as of host cell cysteine proteases implicated in the parasite-induced cell death [13]. Alternatively or in addition, the target(s) of E64 may include members of the parasite serine repeat antigen (SERA) family, which are expressed in mature stages of blood schizonts [14], [15], [16], [17]. SERA proteins may play a role in egress [18], [19], and some of them have E64-sensitive cysteine protease activity [15]. In blood stages some or most SERA proteins are substrates of a conserved *Plasmodium* subtilisin-like serine protease called SUB1 that is discharged from specialised secretory organelles called exonemes into the PV lumen minutes before egress [20], [21], [22], [23]. SUB1 cleaves the SERA proteins to release their central papain-like domain [15], [20]. SUB1-mediated cleavage of *P. berghei* SERA3 (PbSERA3) has been shown to activate its protease activity [15], suggesting that one important role of SUB1 may be to initiate a protease cascade that leads to egress. Discharge of SUB1 into the PV also allows it to modify several other important merozoite proteins, including the major glycolipid-anchored merozoite surface protein MSP1 [24], [25], which is thought to act as an erythrocyte binding ligand [26], [27], [28]. SUB1 therefore likely plays a central role in both development and egress of blood stage schizonts. Intriguingly, whereas both MSP1 and members of the SERA protein family are expressed in liver stage schizonts [9], [11], it is not known whether SUB1 is expressed in liver stages, or whether it has a similarly important role in maturation and release of liver stage merozoites.

The study of genes in liver stages that are essential during the asexual erythrocytic phase of the life cycle requires an inducible or stage specific system for gene disruption. The site specific recombinase Flp is currently the only validated system [29] to knock out or knock down essential genes in liver stages. A panel of highly efficient deleter lines expressing a thermosensitive variant of the recombinase, FlpL, under different sporozoite specific promoters is now available and these have been used to characterise essential malarial gene functions in liver stages, including that of MSP1 [30] and the parasite cyclic GMP (cGMP)-dependent protein kinase, PKG [31]. To control a gene through Flp or FlpL it is necessary to introduce two 34 bp flippase recognition target (*FRT*) sites into the genome such that they flank a crucial part of the target gene, which becomes excised when Flp is expressed, thereby inactivating the gene of interest.

Placing *FRT* sites in a genetic modification vector remains a major challenge. To achieve a complete gene knock out upon activation of Flp, it would be desirable to flank the entire target gene with *FRT* sites. This requires large allelic exchange vectors with at least one very long homology arm comprising the target gene plus an additional 1 kb or more of upstream homologous sequence to achieve genomic integration of the *FRT* site most distant to the selection cassette. Constructing such large vectors in *E. coli* can be difficult, or even impossible, due to the high (>77%) AT content and repetitive nature of genomic DNA (gDNA) of most *Plasmodium* species, which causes instability in circular high-copy plasmids. Recently a *P. berghei* gDNA library with high sequence integrity, relatively large inserts (averaging ∼9.0 kb) and covering now >85% of genes was generated in a linear, low copy plasmid in *E. coli* [32]. Here we present molecular tools and protocols that exploit the efficiency, speed and robustness of recombinase mediated engineering in *E. coli* to convert a gDNA library clone with a 9.6 kb genomic insert containing the *P. berghei sub1* (*pbsub1*) gene into a complex allelic exchange vector for the Flp mediated deletion of the entire *pbsub1* gene. We generate a conditional knock out parasite in which we examine the expression and function of SUB1 in liver stages of *P. berghei*. We show that SUB1 is expressed in subcellular organelles of the liver stage schizont that resemble exonemes, and that expression of the protease is indispensable for completion of the liver stage of the parasite life cycle. Unexpectedly, parasites lacking SUB1 exhibit a defect in development prior to egress, indicating a hitherto unappreciated role for SUB1 in intracellular parasite growth.

## Results

### PbSUB1 is expressed in liver stage schizonts but not in sporozoites

A previous transcriptomic and proteomic analysis of the rodent malaria species *P. yoelii* indicated the presence of *P. yoelii sub1* mRNA in liver stages but detected no SUB1 protein by mass spectrometry [33]. However, mass spectrometric analysis of blood-stage schizonts of the human malaria pathogen *P. falciparum* has detected only between 1 and 8 peptides [34], [35], [36], suggesting that – as with many enzymes - SUB1 is likely a poorly abundant constituent of the total proteome. To address the question of whether SUB1 is expressed in *P. berghei* liver stages, we produced a rabbit polyclonal antibody specific for the catalytic domain of *P. berghei* SUB1 (PbSUB1). Examination of *P. berghei* blood stage schizont extracts by Western blot using the antibody produced signals likely corresponding to the full-length and processed (mature) forms of PbSUB1 (Supplemental Figure S1A in Text S1), by analogy with the maturation profile previously observed with recombinant forms of SUB1 from *P. berghei* and three other *Plasmodium* species [37], [38], [39]. The anti-PbSUB1 antibodies were then used to examine *P. berghei* liver stage EEFs by immunofluorescence assay (IFA). Hepatoma cells infected with sporozoites of a drug selectable marker-free transgenic *P. berghei* clone that constitutively expresses GFP [40] were fixed 64 h post infection and probed with the antibodies. As shown in Figure S1B in Text S1, a punctate signal was obtained that is highly reminiscent of the exoneme-specific pattern previously observed in *P. falciparum* blood stage schizonts probed with polyclonal or monoclonal antibodies (mAb) against *P. falciparum* SUB1 [20], [22]. This suggested that PbSUB1 may be localised in similar subcellular organelles in liver schizonts.

**Figure 1:**
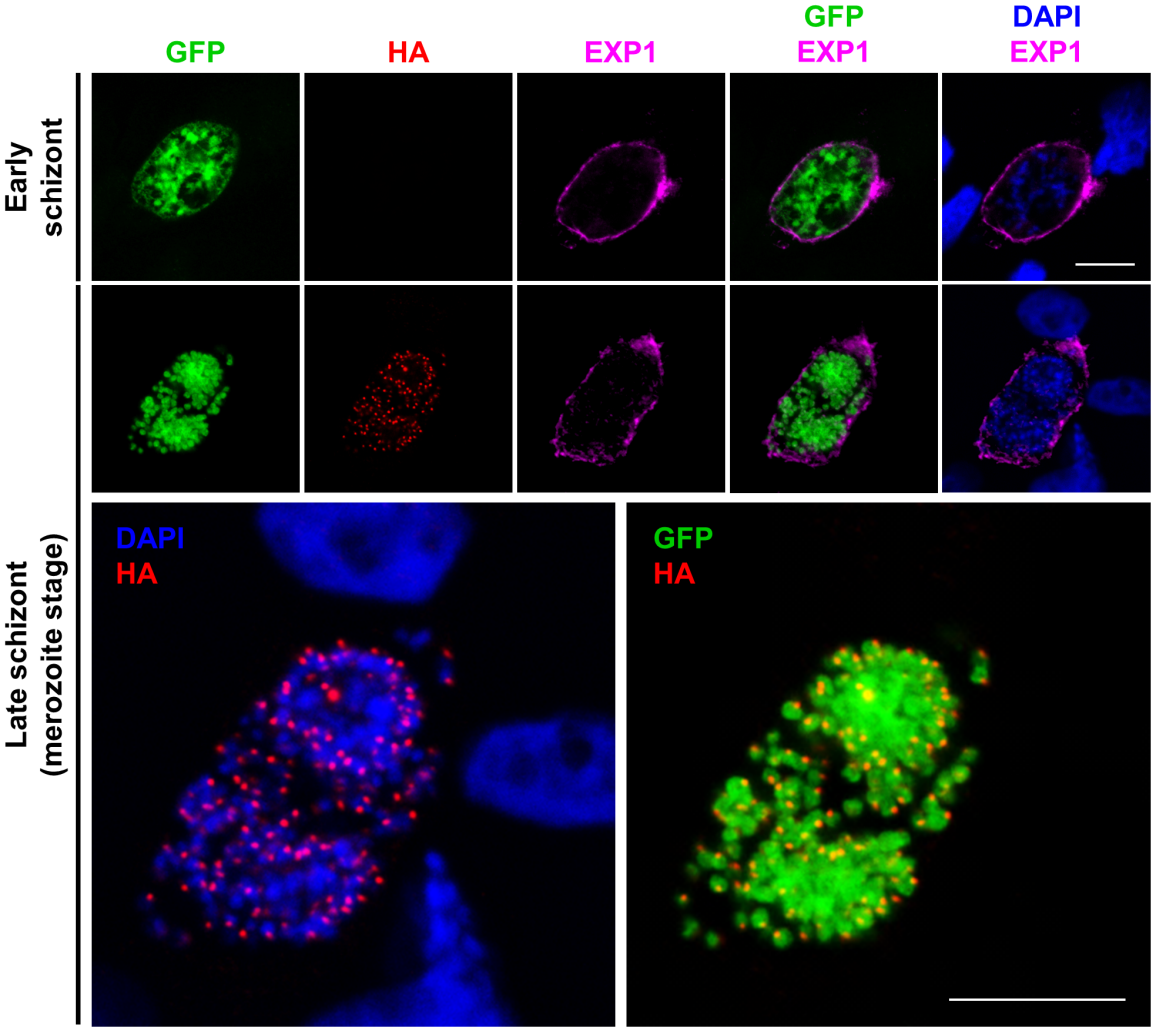
PbSUB1 is expressed in exoneme-like subcellular organelles in mature liver stage schizonts. HepG2 cells infected *in vitro* with sporozoites of the PbSUB1-HA clone were fixed and examined by IFA, probing with an anti-HA mAb, or anti-GFP antibodies, or antibodies against the PVM protein EXP1. Nuclei were counterstained with 4,6-diamidino-2-phenylindol (DAPI, blue). No HA-specific signal was detected in early schizonts (top row) in which formation of individual merozoites is not yet visible. The images on the second row are shown at higher magnification below in order to better visualize the relative localization of the PbSUB1-specific signal relative to the parasite nuclei and cytosol. Scale bar, 10 µm.

To further test this interpretation, we generated a *P. berghei* line expressing epitope-tagged PbSUB1 (called PbSUB1-HA) using homologous recombination to modify the endogenous *pbsub1* gene (PBANKA_110710) in the GFP-expressing parasite background (Figure S2 in Text S1). Western blot analysis of blood stage schizont extracts from PbSUB1-HA parasites using an anti-HA mAb detected a strong double band migrating slightly more slowly than that detected by the anti-PbSUB1 rabbit antibodies (Figure S3A in Text S1), consistent with the expected small increase in mass of the epitope-tagged PbSUB1 as a result of its fusion to the HA epitope tag. In contrast, Western blot analysis of PbSUB1-HA salivary gland sporozoite extracts with the anti-HA antibody, or with the anti-PbSUB1 antisera, detected no specific signal (not shown). IFA analysis of mature blood stage (Figure S3B in Text S1) or mature liver stage (Figure 1 and Figure S4 in Text S1) PbSUB1-HA schizonts with the anti-HA mAb again produced a clear punctate signal. The foci were associated with but distinct from individual merozoite nuclei, and again similar to the exoneme-specific signal previously observed in *P. falciparum* blood stage schizonts. No PbSUB1-HA IFA signal was detected in salivary gland sporozoites or early liver stage schizonts (Figure S3C in Text S1, Figure 1 top row and Figure S4 in Text S1) or at the cytomere stage when the parasite plasma membrane is just beginning to invaginate to surround groups of nuclei (Figure S5 in Text S1). Collectively, these results convincingly demonstrate that PbSUB1 is not expressed in sporozoites or early EEFs, but is expressed in mature liver stage schizonts, where it likely accumulates in subcellular organelles similar to the exonemes previously described in blood stage schizonts.

### Transgenic parasite clones for Flp mediated conditional deletion of *pbsub1*

**Figure 2:**
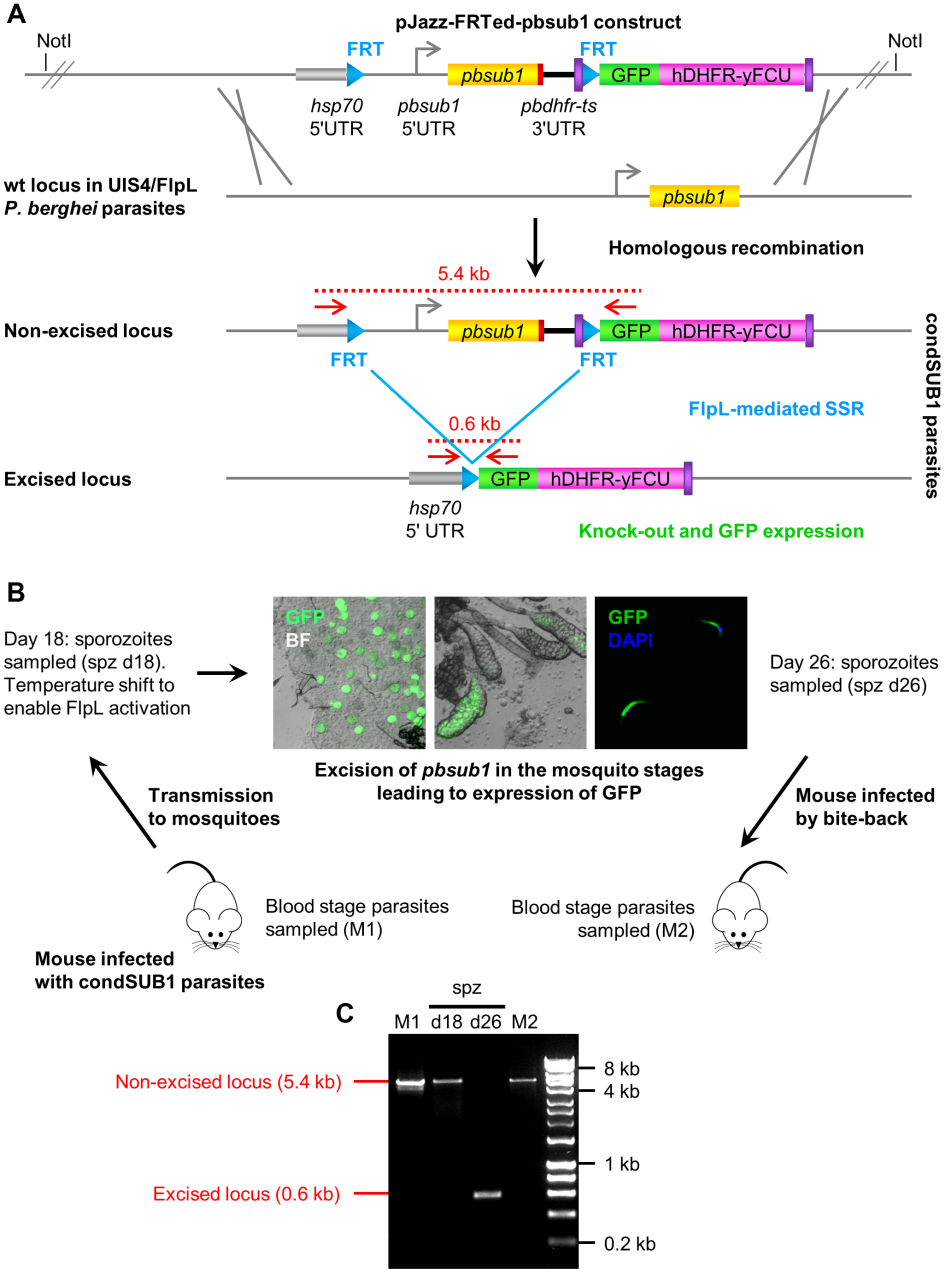
Insect stage-specific conditional deletion of the *pbsub1* gene blocks the transition from salivary gland sporozoite to subsequent asexual blood stages. (A) Double crossover homologous recombination strategy to simultaneously flirt and epitope-tag the *pbsub1* gene, whilst also introducing a GFP reporter for successful gene excision. A ∼10 kb genomic DNA library clone containing the *pbsub1* gene was modified by recombineering and Gateway technology to place an *FRT* site ∼2.3 kb or ∼1.8 kb upstream of the *pbsub1* gene and directly downstream of an inserted *P. berghei hsp70* promoter. A second downstream *FRT* site was inserted in frame with a GFP reporter coding sequence so that, upon excision, the *hsp70* promoter drives expression of GFP. The final transfection constructs (pJazz-FRTed-pbsub1 and pJazz-FRTed-pbsub1_short_), which also contained a hDHFR-yFCU positive-negative selectable marker cassette, were transfected into the *P. berghei* UIS4/FlpL clone [30] and integrant parasite clones obtained, called condSUB1 (clones A and B) and condSUB1_short_. (B) Mosquitos fed on mice infected with condSUB1 clone A parasites were subjected to a temperature shift 18 days post transmission to ensure optimal activity of the FlpL recombinase. Oocysts, salivary glands and sporozoites from these insects displayed strong GFP expression at 26 days, when the insects were allowed to feed on naive mice (bite-back). Resulting blood stage parasites were collected. (C) PCR analysis of blood stage condSUB1 parasites from mouse 1 (M1) and mouse 2 (M2), as well as sporozoites isolated 18 or 26 days following transmission. The primers used (F_out_hsp70 and R_out_GFP; red arrows in panel A, see Table S1 for sequences) produce a 5.4 kb amplicon from the non-excised modified *pbsub1* locus, or a 600 bp product from the excised locus (panel A). The results indicate that, whereas excision occurred highly efficiently in the 26 day sporozoites to the degree that the non-excised locus was undetectable by PCR, only residual non-excised parasites were capable of establishing a blood stage infection in the bite-back mice. Note that since smaller PCR products are generally produced more efficiently than large amplicons, the PCR result likely exaggerates the degree of excision in the 26 day sporozoites. Microscopic examination of dissected d26 condSUB1 clone A sporozoites showed that the proportion of GFP-positive sporozoites in these experiments was usually ∼90% (data not shown).

In our previous work on *P. falciparum* SUB1 [20] we were unable to obtain viable parasites in which the *pfsub1* gene was disrupted, suggesting an essential role in blood stages. Attempts to disrupt the *pbsub1* gene in *P. berghei* blood stages similarly failed (S. Yeoh, R. Tewari, O. Billker and M. Blackman, unpublished), suggesting that PbSUB1 too is indispensable in the erythrocytic parasite life cycle. To study the role of the *pbsub1* gene in liver stages we therefore decided to exploit a recently-described conditional deletion approach [30], [41] in which stage-specific expression of the *Saccharomyces cerevisiae* site-specific recombinase Flp (or its thermosensitive variant FlpL) is employed to disrupt a target gene in the late mosquito stages of the parasite life cycle. Working from a *P. berghei* genomic DNA library clone from the *Plasmo*GEM resource (http://plasmogem.sanger.ac.uk/) containing 9.6 kb of the *pbsub1* locus and neighbouring genes, we constructed allelic exchange vectors designed to flank the entire *pbsub1* coding sequence with *FRT* sites, while at the same time inserting a C-terminal HA epitope tag into *pbsub1* (Figure 2A). The 5′ *FRT* site was introduced into one of two alternative positions in the large upstream intergenic region together with a constitutive promoter sequence of the *P. berghei hsp70* gene. A promoterless GFP coding sequence was positioned immediately downstream of the second *FRT* site (Figure 2A and Figure S6 in Text S1). Precise placement of the *FRT* sites and other exogenous sequence both upstream and downstream of *pbsub1* was achieved in 4 steps by recombinase mediated genetic engineering in *E. coli*, using both the improved Red/ET recombinase system of lambda phage [42] and transient expression of Flp as described in Supplemental Methods and Figure S6 in Text S1. The constructs were designed such that, following integration by ends-out homologous recombination into the parasite genome, correct recombinase-mediated excision of the sequence lying between the *FRT* sites (which included the epitope-tagged *pbsub1* gene) would reposition the GFP reporter adjacent to the *hsp70* promoter, driving constitutive GFP expression only in those parasites in which deletion of the *pbsub1* gene had occurred (Figure 2A and Figure S6 in Text S1). To prevent the *FRT* site from interfering with initiation of translation [30], the start codon for GFP expression was placed just upstream of the 5′ *FRT* site, such that after excision the *FRT* sequence would be translated into a 12 residue N-terminal extension of GFP. The final constructs, called pJazz-FRTed-pbsub1 and pJazz-FRTed-pbsub1_short_ (which differed only in the placement of the 5′ *FRT* site at either ∼2.3 kb or ∼1.8 kb respectively upstream of the start ATG of the *pbsub1* gene) contained ∼7 kb and 700 bp regions of homology respectively at their 5′ and 3′ ends for homologous integration into the *P. berghei* genome.

The constructs were transfected into the *P. berghei* UIS4/FlpL deleter clone [30] which expresses FlpL under control of the sporozoite stage-specific *uis4* promoter. Transfected parasites were expanded under pyrimethamine treatment and cloned by limiting dilution to obtain 2 independent parasite clones (named condSUB1 clone A and B) transfected with the pJazz-FRTed-pbsub1 construct, and a single parasite clone transfected with the pJazz-FRTed-pbsub1_short_ construct (called condSUB1_short_). The expected homologous integration event in each of the parasite clones was confirmed by diagnostic PCR, Southern blot and pulse-field gel analysis (Figure S6 in Text S1).

### PbSUB1 is essential for completion of the parasite pre-erythrocytic stages

The condSUB1_short_ and condSUB1 parasite clones did not express GFP, as expected, and exhibited no growth phenotype in blood stages (data not shown), indicating that the modifications to the *pbsub1* locus resulting from integration of the targeting constructs did not affect parasite viability. To initially assess stage-specific deletion of the modified *pbsub1* gene in the condSUB1 clones, *Anopheles stephensi* mosquitoes fed on mice infected with the condSUB1 clone A parasites were subjected to a temperature shift to 25°C at 18 days following transmission in order to enhance activity of FlpL. At day 26 post-transmission dissected midguts, salivary glands and salivary gland sporozoites were examined microscopically. GFP expression was observed in 65±19% of the oocysts as well as in the majority of sporozoites recovered from the condSUB1 clone A-infected insects, consistent with the expected FlpL-mediated excision event (Figure 2B). There was some variation between individual experiments (n = 4), but visual microscopic examination of the isolated day 26 condSUB1 sporozoites showed that the proportion of GFP-positive sporozoites was usually ∼90% (though this varied somewhat in subsequent experiments; see below), similar to previous findings of others using the Flp/*FRT* system in *P. berghei* [30], [41], [43]. Analysis by genotyping PCR of sporozoites collected at day 18 and day 26 following transmission (Figure 2C) confirmed efficient, time-dependent excision of the flirted *pbsub1* gene, with undetectable levels of the non-excised *pbsub1* locus in the day 26 sporozoites. To examine the behaviour of the *pbsub1*-deficient parasites throughout their lifecycle, condSUB1-infected mosquitoes were allowed to feed on naïve mice at 26 days following transmission (‘bite-back’ infection), by which point most of the sporozoites observed in the insect salivary glands were expressing GFP. The bite-back mice were monitored for the appearance of blood-stage parasites, which were then recovered and analysed by genotyping PCR. As shown in Figure 2C, despite the predominance of the excision event in the previous mosquito stages, only the non-excised *pbsub1* locus was detectable in the erythrocytic parasites that appeared in the bite-back mice (observations from n = 7 independent experiments, each using 3–5 mice). These parasites did not express GFP (not shown). Identical results were obtained in an independent experiment with the condSUB1 clone B as well as the condSUB1_short_ parasites (Figure S7 in Text S1). These results strongly suggested that those sporozoites in which the *pbsub1* gene had been deleted were incapable of successfully establishing a blood stage infection.

### PbSUB1-deficient sporozoites efficiently invade hepatocytes and establish a liver stage infection

**Figure 3:**
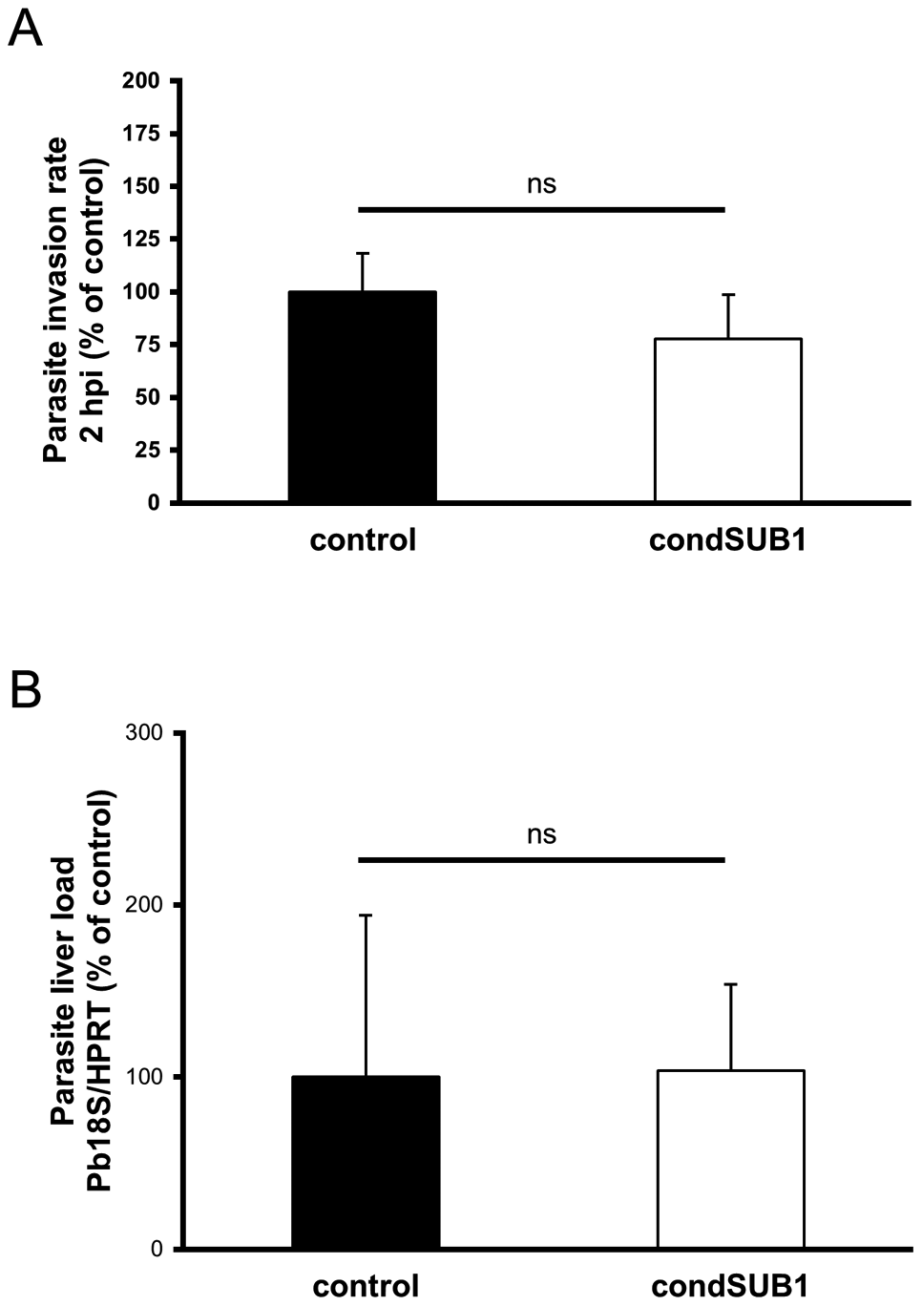
Excised condSUB1 sporozoites display no defect in hepatocyte invasion or capacity to establish a liver stage infection. (A) Deletion of the *pbsub1* gene does not affect the invasive capacity of sporozoites *in vitro*. HepG2 cell monolayers were infected with equal numbers of condSUB1 (∼85% excised) or control UIS4/FlpL-F sporozoites. 2 h post infection (2 hpi), cell monolayers were washed, fixed, and processed for in-out staining with anti-CSP and anti-GFP antibodies and the nuclear stain Hoechst 33342. Quantification of intracellular parasites was performed by Cellomics automated microscopy and image analysis software. Results are expressed as the mean ± SD (3 experiments, each using n = 3–7 replicate wells) of the number of infected cells as a proportion of those infected with control parasites. There is no significant difference between the means (Student's *t*-test). (B) Deletion of *pbsub1* does not reduce levels of liver infection *in vivo*. Mice were infected by intravenous inoculation of 20,000 condSUB1 (∼85% excised) or control sporozoites. After 40 h, total RNA was prepared from whole livers and levels of 18S ribosomal parasite RNA and mouse hypoxanthine guanine phosphoribosyltransferase (HPRT) mRNA quantified by qRT-PCR. Relative amounts of RNA were calculated using the ABI Prism 7000 SDS 1.2.3 Software, and normalised against the expression levels of the mouse housekeeping gene HPRT. Data are expressed as the mean ± SD (3 experiments each using n = 3–5 mice). There is no significant difference between the means (Student's *t*-test).

To gain more insight into the nature of the defect resulting from *pbsub1* deletion, we next examined whether the inability of PbSUB1-deficient sporozoites to establish a blood stage infection was a result of compromised hepatocyte invasion, although we considered this unlikely given our previous evidence that PbSUB1 is not expressed in sporozoites. To investigate this, we incubated hepatoma cells *in vitro* with 26 day condSUB1 clone A or clone B sporozoites and assessed their capacity to invade the cells. For this we used a modified differential staining assay [44] that distinguishes extracellular (i.e. residual cell surface-bound) sporozoites from intracellular parasites, combined with automated microscopy and high content image analysis software. Extracellular sporozoites were detected with an antibody against the circumsporozoite protein (CSP) on the parasite surface, whilst an antibody against GFP was used to detect all excised condSUB1 sporozoites. As a control for these assays we used sporozoites of the *P. berghei* UIS4/FlpL-F clone [41], which constitutively expresses GFP under the same *hsp70* promoter as used in the condSUB1 and condSUB1_short_ clones. These sporozoites were also obtained from insects that had been placed at 25°C at 18 days following transmission. As shown in Figure 3A, no significant differences were observed in the proportions of GFP-positive infected host cells detectable 2 h following addition of control or condSUB1 sporozoites, indicating equivalent invasive capacity. This observation was confirmed under *in vivo* conditions by using qRT-PCR to quantify parasite liver loads following infection of mice with condSUB1 or control sporozoites. As shown in Figure 3B, there was no significant difference in parasite liver loads measured 40 h after intravenous inoculation of 20,000 condSUB1 or UIS4/FlpL-F sporozoites. These results showed that condSUB1 sporozoites are fully competent to initiate and establish a liver infection.

### PbSUB1-deficient parasites are impaired in hepatic schizont development and merozoite formation

**Figure 4:**
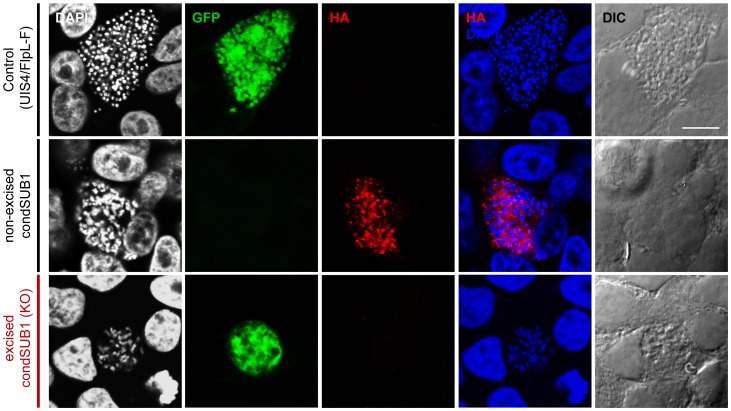
Expression of epitope-tagged PbSUB1 in liver stage schizonts of condSUB1 clone A, and detection of a developmental defect associated with *pbsub1* excision. HepG2 cells infected *in vitro* with sporozoites of the indicated clones were fixed at 64 h post infection and probed with anti-GFP (green) or anti-HA (red) antibodies. DAPI-stained nucleic acids are shown in blue, whilst differential interference contrast (DIC) was used to obtain bright field images of the infected cells. The non-excised condSUB1 liver stage schizonts displayed the expected punctate HA signal, indicating the expected epitope tagging of PbSUB1. No HA signal was associated with the excised condSUB1 EEFs; however, mature schizont stages displaying merozoite production could not be found, suggesting a defect in liver stage development. Scale bar, 10 µm.

Having determined that *pbsub1*-null sporozoites display no invasion phenotype *in vitro* or *in vivo* and are able to efficiently initiate intrahepatic growth, we next addressed whether the transmission defect observed in the excised condSUB1 parasites was due to a defect in subsequent liver-stage replication. Development of hepatic EEFs comprises a well-described set of morphological transitions, in which an early schizont gradually increases in size and passes through cytomere and merozoite formation stages before rupture of the PVM to allow release of the mature merozoites into the host cell cytosol. To initially assess expression of epitope-tagged PbSUB1 in the condSUB1 parasites and to attempt to confirm loss of PbSUB1 expression upon excision, we used IFA to compare the GFP-positive (excised) and GFP-negative (non-excised) EEFs obtained following infection of hepatoma cell cultures with condSUB1 clone A or B sporozoites, using antibodies against GFP and the HA epitope tag fused to PbSUB1. Whilst mature GFP-negative (non-excised) condSUB1 liver stage schizonts displayed the expected punctate IFA signal, as observed previously with the PbSUB1-HA clone, we were able to detect only immature forms of GFP-positive (excised) condSUB1 EEFs (Figure 4), with no mature forms visible. This unexpected result was explained by subsequent findings described below.

**Figure 5:**
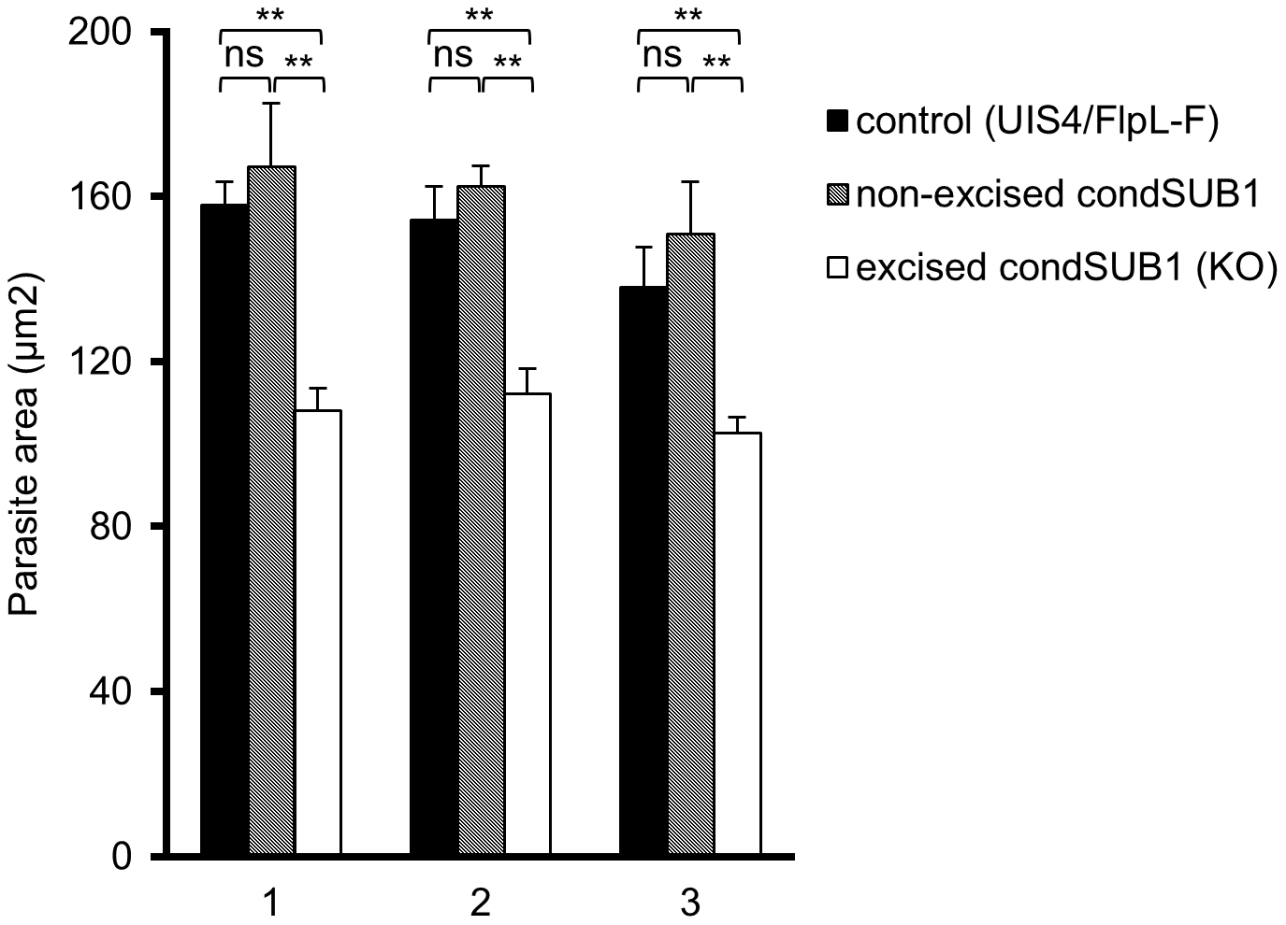
PbSUB1-deficient EEFs are slightly smaller than non-excised or control EEFs. Shown are mean parasite surface area values obtained by IFA analysis of cytoplasmic staining obtained by probing with antibodies against PbHSP70 48± SD. Data are presented from three independent experiments (1–3, X axis), involving analysis of a total of >2,500 condSUB1-infected cells and ∼4,000 UIS4/FlpL-F-infected cells. Discrimination between the non-excised and excised (PbSUB1-deficient) condSUB1 parasites was by the presence of GFP expression. Images were acquired using a Cellomics ArrayScan VTI HCS Reader and analysed using the SpotDetector BioApplication as described in Materials and Methods. For each comparison, Students *t*-test was performed and the threshold alpha of the p value was set to 0.001 (**). In these experiments, the proportion of excised condSUB1 sporozoites in the preparations used to infect the hepatocytes was ∼80%, whilst the proportions of non-excised EEFs in the infected cultures were calculated as 29±5.5% at 28 h post-infection and 33±1.6% at 48 h post infection.

To investigate the intrahepatic development of PbSUB1-deficient parasites, the size of GFP-positive condSUB1 EEFs in infected hepatoma cell cultures was analysed at 28 h and 48 h post infection, comparing them with non-excised condSUB1 and control UIS4/FlpL-F EEFs using automated microscopy and image analysis software. Parasite identification in this case was achieved using antibodies to the *P. berghei* HSP70 heat-shock protein, whilst GFP expression was used to discriminate excised PbSUB1-deficient (GFP-positive) condSUB1 parasites from the minority of non-excised condSUB1 parasites. No significant differences in EEF dimensions were seen at the 28 h time point (data not shown). However, as shown in Figure 5, a small but significant reduction in the mean surface area (28±3%) of the PbSUB1-deficient parasites was observed at 48 h post infection compared to both controls, indicating a subtle defect in schizont development.

**Figure 6:**
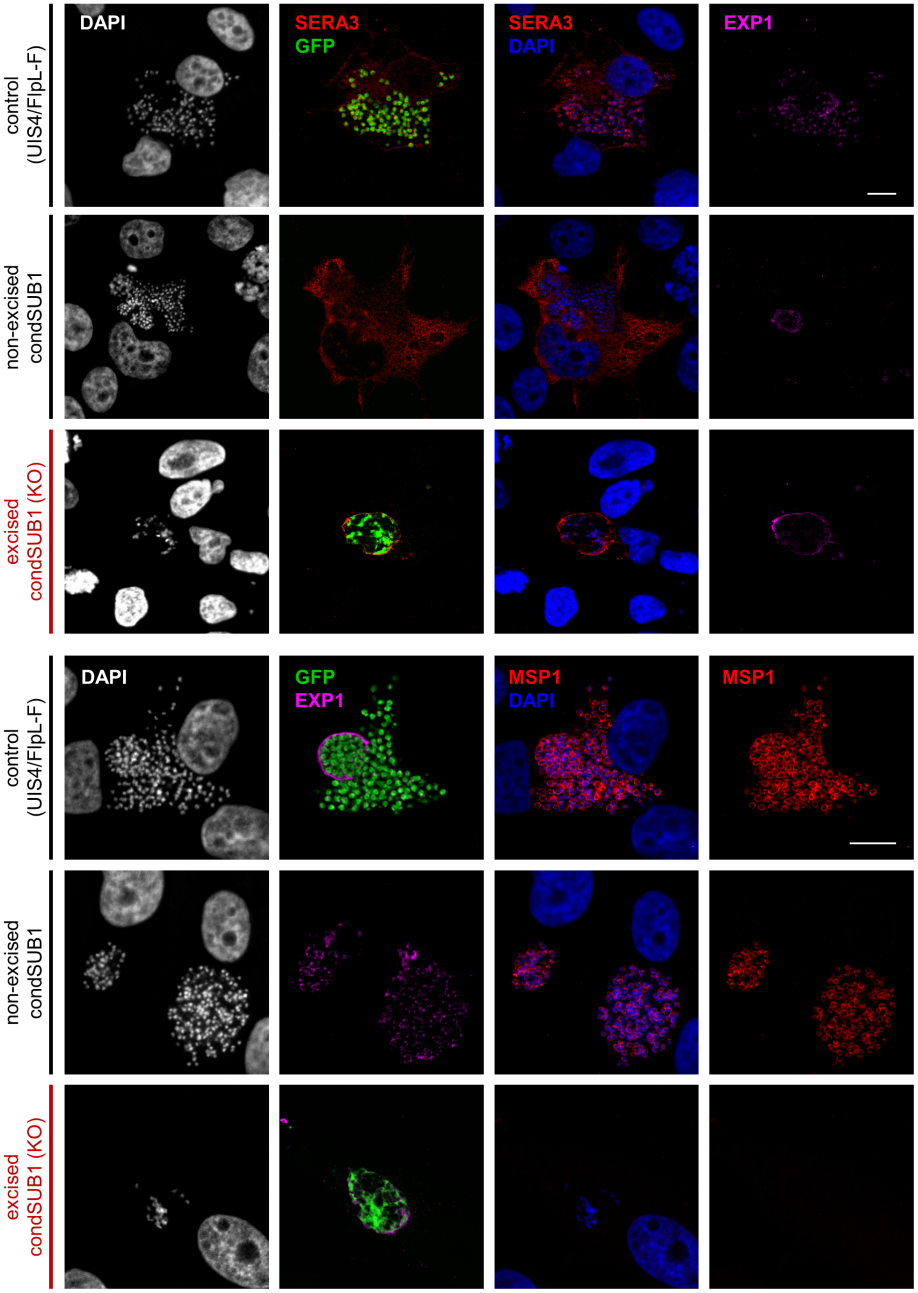
PbSUB1-deficient liver stage schizonts are defective in development. IFA analysis of hepatoma cells 60–64 h post infection with excised or non-excised condSUB1 clone A sporozoites, or control UIS4/FlpL-F sporozoites. Antibodies to the PV protein PbSERA3 (red), GFP (green), the plasma membrane marker MSP1 (red), or the PVM marker EXP1 (pink) were used to probe the samples. Nucleic acids were stained with DAPI (white or blue). Both merozoite production and PVM rupture were defective in the excised (PbSUB1-deficient) condSUB1 EEFs. Identical results were obtained for the condSUB1 clone B parasites (not shown). Note that the non-excised condSUB1 parasites do not express GFP. Scale bar, 10 µm.

**Figure 7:**
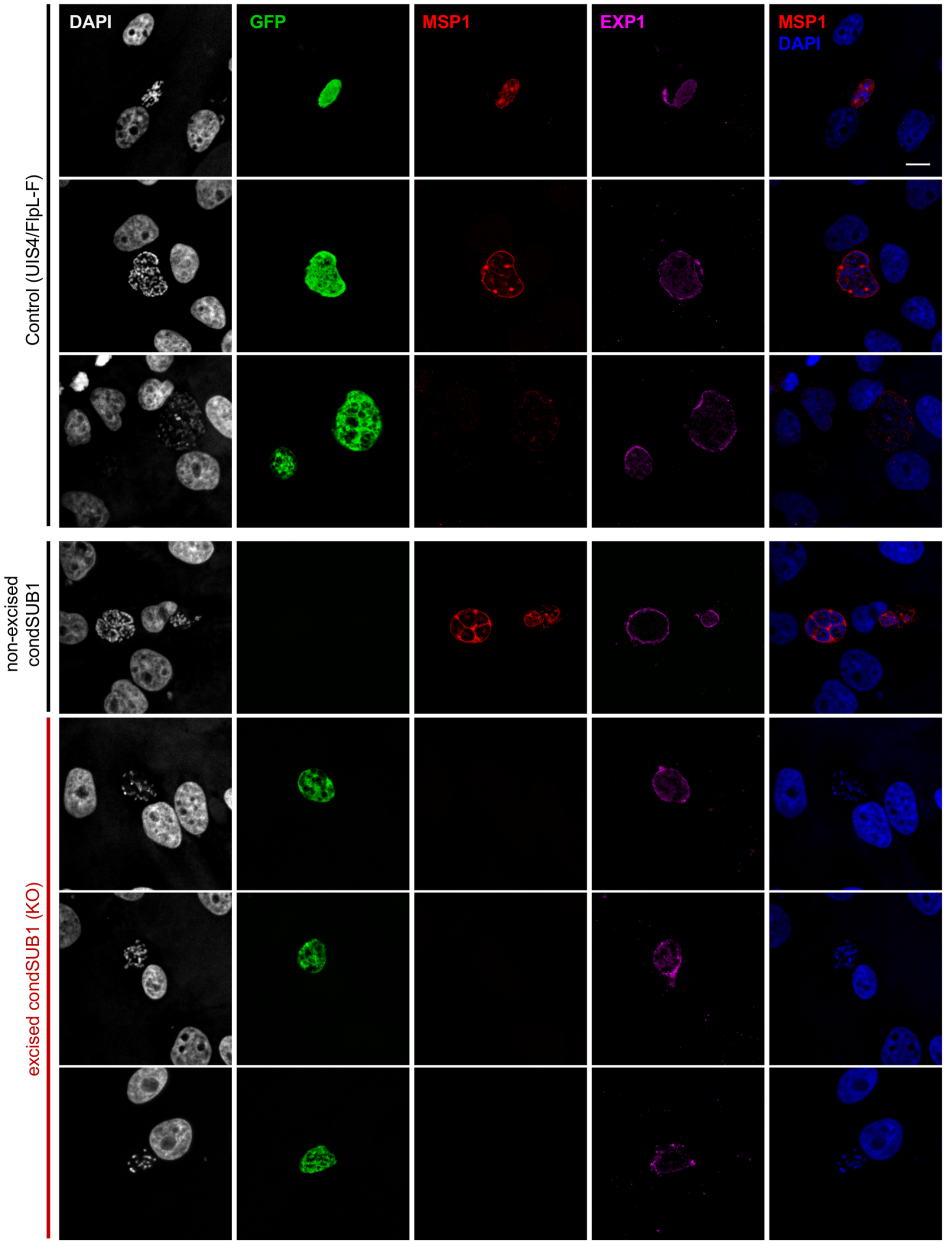
Onset of MSP1 expression is ablated in PbSUB1-deficient EEFs. HepG2 cells infected with sporozoites of the indicated clones were fixed 48–56 h post infection and probed with antibodies against MSP1 (red), EXP1 (pink), or GFP (green). DAPI-stained nucleic acids are shown in white or blue. MSP1 expression was undetectable in the excised (GFP-expressing) condSUB1 EEFs. Scale bar, 10 µm.

For a more detailed analysis of this phenotype, we investigated the appearance throughout EEF maturation of three parasite marker proteins with distinct subcellular locations. Starting from 24 h post invasion, infected hepatoma cells were examined by IFA using antibodies against the PVM protein EXP1, the soluble PV protein PbSERA3 (a late liver stage marker expressed from cytomere stage onwards that is eventually released into the host cell cytosol; [11]), and the plasma membrane protein MSP1 (present from cytomere stage onwards and involved in the formation of hepatic merozoites [30]). At time points up to and including cytomere stage, expression and localisation of EXP1 and PbSERA3 was normal in the PbSUB1-deficient parasites (Figure S8 in Text S1). However, at very late time points (from around 64 h onwards), whilst normal rupture of the PVM and associated release of PbSERA3 into the host cell cytoplasm was evident in the majority of the control infected cells, it was only very rarely detected in the PbSUB1-deficient parasites (Figure 6). Equally strikingly, whereas control parasites displayed as expected a clear MSP1 signal from cytomere stage onwards, which subsequently translocated to surround individual merozoites, the majority of the PbSUB1-deficient parasites completely lacked a detectable MSP1 signal (Figure 6 lower panels and Figure 7) and showed no signs of correct merozoite formation. Multiple nuclei were observed in the early PbSUB1-deficient schizonts (Figure 7), indicating normal nuclear replication, but in later stages many of the schizont nuclei appeared condensed and abnormal.

**Figure 8:**
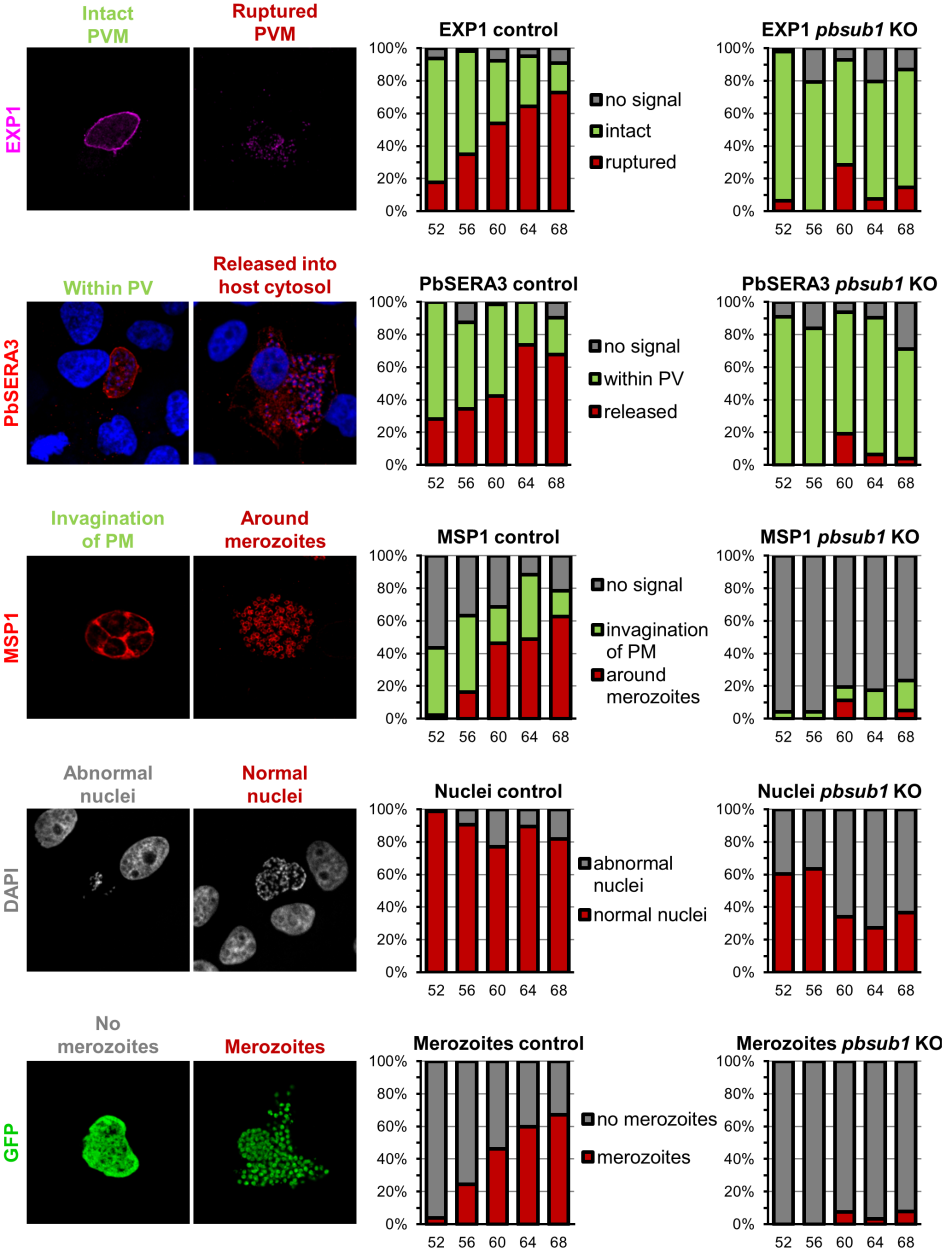
Merozoite formation, PV rupture and the expression profile of MSP1 is abnormal in maturing PbSUB1-deficient liver stage schizonts. Comparative IFA of EEFs obtained following infection of hepatoma cells with either control UIS4/FlpL-F sporozoites or excised condSUB1 (*pbsub1* KO) clone A sporozoites. Antibodies or dyes used to probe the samples are indicated on the left, whilst typical representative morphologies obtained are shown in the associated microscopic images. Each bar on the histograms refers to a total of 40–60 randomly-selected images which were acquired at each of 5 time points (52, 56, 60, 64 or 68 h) post infection. The classification of these images based on morphology is indicated by the height of the coloured sections of each bar. The colour code for each different morphology is indicated alongside the sets of histograms.

To produce a quantitative description of these observations, we used confocal microscopy in two independent experiments to categorise a total of 40–60 individual parasitised hepatoma cells at each of several time points following infection with either the excised condSUB1 parasites or the control UIS4/FlpL-F parasites (Figure 8). This analysis confirmed no significant difference in expression and localisation of the 3 marker proteins prior to 52 h post infection. Subsequent to this, however, the PbSUB1-deficient parasites began to display clear differences in the levels or expression pattern of the marker proteins, culminating in a nearly complete absence of formation of daughter merozoites.

### PbSUB1-deficient parasites are defective in merosome formation

Merozoite egress from infected hepatocytes is via extrusion of merosomes, vesicles filled with mature invasive merozoites. To address the consequences of PbSUB1 depletion specifically on merosome formation, hepatoma cell monolayers infected *in vitro* with condSUB1 or control UIS4/FlpL-F sporozoites were cultured to allow complete parasite development and then cell supernatants were repeatedly harvested between 62–70 h post infection. At these time points, the supernatants normally contain non-adherent rounded-up infected cells as well as merosomes (Figure S9 in Text S1). The detached cells were collected, fixed in suspension, gently centrifuged and processed for IFA, then counted. As shown in Figure S10 in Text S1, a clear deficiency in formation of detached cells and merosomes was observed in the case of the GFP-positive (excised) condSUB1 parasites. In contrast, the fraction of condSUB1 parasites in which *pbsub1* excision had not occurred (and which therefore did not express GFP) produced ∼10-fold more merosomes despite representing only ∼20% of the input condSUB1 parasite population, providing an internal control demonstrating that *pbsub1* deletion rather than the presence of the modified *pbsub1* locus was responsible for the defect in merosome formation. To exclude the possibility that the excised condSUB1 parasites might simply exhibit a delay in merosome formation, cultures were further monitored for up to 85 h post infection; however, even following such prolonged culture, no GFP-positive merosomes were observed in the condSUB1-infected hepatoma cell supernatants (data not shown). Injection of the condSUB1 merosome preparations into naïve mice resulted in a blood-stage infection which contained only non-excised parasites (data not shown), mirroring the bite-back data described in Figure 2 and Figure S7 in Text S1. These results unambiguously confirmed that PbSUB1 is required for completion of liver stage development and formation of infectious liver stage merozoites.

## Discussion

Studying essential blood stage genes in *Plasmodium* liver stages requires regulatable genetic approaches. A tetracycline repressible transactivator has recently been shown to allow dynamic gene regulation in *P. berghei* blood stages *in vivo* and in liver stages *in vitro* [45], but it remains to be tested whether it can be used successfully on essential liver stage genes. While the potential to use the same transgenic parasite for studying essential genes at different life cycle stages would clearly be an attraction of a tetracycline regulatable system, the sequence-specific Flp recombinase system applied here already provides a powerful tool to study essential genes specifically in the sporozoite and liver stage. However, precise placement of *FRT* sites has remained a major challenge. In the mouse, target sites for recombinases can often be designed to flank a critical exon excision of which results in effective gene disruption (e.g. [46]). In malaria parasites, in contrast, where genes are relatively short and only around half have introns, flanking the entire protein coding sequence of a gene with *FRT* sites would be a good default strategy. However, the high AT content and repetitive nature of *P. berghei* genomic DNA has until now made it impractical to construct such genetic modification vectors for larger genes, since long genomic fragments are unstable in conventional circular high-copy plasmids in *E. coli*. Matters are further complicated by the observation that *FRT* sites positioned within 100 bp upstream of a start codon can interfere with gene expression in *P. berghei* [30]. As a consequence, recent studies have resorted to excising only the 3′ regulatory sequence of a gene [30], [41], [43], resulting in gene silencing due to destabilisation of the mRNA, rather than achieving a complete gene knock out. Whilst this approach can work well, it is not uniformly successful due to the potential for cryptic polyadenylation sites downstream of the modified gene that act to stabilise the mRNA [47], [48]. Furthermore, it is a drawback of this strategy that it sacrifices the key advantage of recombinases in providing the very tight regulation that stems from complete removal of a target gene.

We here have demonstrated that a large genomic insert from a *P. berghei* genomic DNA library in a low copy linear pJAZZ vector can be manipulated successfully within *E. coli* using sequence-specific and lambda Red/ET recombinases. This approach, which is well established for generating conditional knock out alleles for the mouse (e.g. [46]), allows *FRT* sites to be placed almost at will and without the need to manipulate *Plasmodium* DNA by conventional restriction/ligation cloning. We also generated some of the PCR templates and a Gateway donor cassette that can form part of a more generic strategy for turning large gDNA inserts into complex conditional knock out alleles for *P. berghei* genes. In the current study, to ensure positioning of the upstream *FRT* site well away from potentially important flanking promoter elements we chose a position ∼2.3 kb or ∼1.8 kb away from the *pbsub1* ATG start codon, distances substantially larger than most *Plasmodium* promoter sequences mapped to date. We also chose to integrate a strong promoter 5′ to the *FRT* site in the upstream intergenic region of the target gene. This successfully allowed us to use expression of GFP to monitor excision of the *pbsub1* gene in individual liver stage parasites. As a result, in our microscopic analyses of the *pbsub1* knock out phenotype we were able throughout to distinguish excised parasites clearly from the minority of parasites in which excision had failed. While this strategy proved useful in the current study, where a large upstream intergenic region was available, for other genes integrating a long promoter sequence may interfere in unpredictable ways with expression either of the target gene itself, or of a neighbouring gene. In such cases it will be possible to omit the *hsp70* promoter from the PCR amplicon in Step 1, leaving behind only the 34 bp *FRT* site after Step 2. Importantly, with the tools described here, constructs similar to that described can be produced and quality-control evaluated within as little as 16 working days. The robustness and speed of recombinase mediated engineering means it can be carried out in continuous liquid culture on 96 well plates. Insertion of an upstream *FRT* site in Steps 1 and 2 in such an optimised protocol could probably be completed within a week. Steps 3 and 4 can be adapted to use our existing pipeline for Red/ET mediated engineering on 96-well plates [32]. Such an optimised recombinase based strategy would greatly ease the generation of complicated conditional alleles and the study of essential genes in liver stages.

Excision of the flirted *pbsub1* gene, as indicated by expression of the GFP reporter in our transgenic parasites, occurred efficiently at the oocyst stage despite expression of FlpL under control of the *uis4* (maximally upregulated in infectious sporozoites) promoter. We were not overly surprised by this observation, since although this promoter is maximally active in salivary gland sporozoites [49] we are not aware of any previous evidence that it is entirely ‘off’ in oocysts. It is important to note that GFP expression in an oocyst does not imply that *all* the resident sporozoites have undergone excision, only that some have done so. On the other hand it is also conceivable that - given the mode of parasite replication in oocysts, in which sporozoites bud from a syncytial sporoblast containing multiple nuclei [50] - GFP produced by excised parasites could be incorporated into the cytosol of non-excised sporozoites developing within the same oocyst. This could lead to a degree of over-estimation of the excision rate in our study, which may explain the apparent small mismatch between the proportions of GFP-positive (excised) input sporozoites and GFP-negative (non-excised) EEFs observed in some of the *in vitro* hepatocyte infection experiments (e.g. Figure 4). Importantly, the stage specificity of the *uis4* promoter in this system is supported by the fact that the transgenic condSUB1 and condSUB1_short_ parasites showed no growth defect in the asexual blood stages, and no GFP expression was observed in those stages (although this does not rule out low level ‘leaky’ FlpL expression since excision of *pbsub1* in asexual blood stages would likely produce non-viable parasites).

Our finding that hepatocyte invasion, and the early stages of liver stage growth were all normal in the PbSUB1-deficient condSUB1 parasites confirms that PbSUB1 does not play an important role in these phases of the parasite life cycle; indeed, an effect on hepatocyte invasion was not expected anyway since PbSUB1 expression was undetectable in salivary gland sporozoites, either using our polyclonal anti-PbSUB1 antibodies or by epitope-tagging. In contrast, investigation of more mature liver stages of the PbSUB1-deficient parasites revealed a clear defect in merozoite formation. Unexpectedly, this was associated with an apparent absence of expression of the glycolipid-anchored major plasma membrane protein MSP1. MSP1 is an established SUB1 substrate, but our previous studies in blood stages [20], [22] have demonstrated that SUB1-mediated proteolysis of MSP1 requires discharge of SUB1 from exonemes, consistent with the topology of MSP1 on the merozoite surface where it is effectively a component of the PV lumen. We therefore had no *a priori* reason to predict that an absence of SUB1 should affect MSP1 expression or trafficking. Similarly unexpected was the observation that the MSP1 expression defect in PbSUB1-deficient parasites, as well as a small but significant effect on the size of the intracellular EEFs, was evident well before the point at which expression of PbSUB1 was detectable by IFA. We interpret this result as indicating that PbSUB1 plays an important role in EEF development before significant accumulation of the protease in exonemes. It is conceivable that an absence of SUB1 impacts on EEF exoneme biogenesis, or even that SUB1 plays a general processing role in parasite protein trafficking, analogous to that of certain members of the subtilisin-like prohormone convertase family [51]. Further work will be required to explore this possibility. Whether these additional roles for SUB1 also operate in blood stages will require the application of a conditional expression strategy suitable for use in *Plasmodium* blood stages, such as the recently-described DiCre system [48], and this work is underway.

MSP1 has previously been shown to be important for merozoite development [30]. It is perhaps unsurprising then, that a block in merosome formation was evident in the PbSUB1-deficient parasites; this is likely a direct result of the defect in merozoite formation. It was also accompanied by a block in PVM rupture. In blood stages, members of the SERA family have been implicated in egress. Many or most blood stage SERA family members are substrates of SUB1 [20], [25], [38], and moreover at least one member of the SERA protein family in *P. berghei*, PbSERA3, undergoes proteolytic processing in liver stages [11]. It is therefore possible that the observed defects in PVM rupture and egress are due to an absence of PbSUB1-mediated processing of PbSERA3 and/or other SERA proteins.

Of the two other liver stage parasite proteins that have previously been implicated in egress using gene disruption approaches - liver-specific protein 1 (LISP1; [52]) and the parasite cyclic GMP-dependent protein kinase (PKG; [31]) – the latter has recently been shown to play a key role in regulating discharge of SUB1 into the PV in blood stage schizonts [22]. Given our definitive evidence here for expression of SUB1 in liver stages and its essential role, it is tempting to speculate that the egress defect observed in the PKG knockout reported by Falae et al. [31] is at least in part due to a resulting block in SUB1 discharge. Future work will focus on the fate of SUB1 in PKG knockout EEFs.

In conclusion, we have combined cutting-edge molecular tools with a conditional gene deletion strategy to obtain the first complete conditional deletion of a liver stage *Plasmodium* gene. The molecular strategies described here will render stage-specific regulation of gene expression in *Plasmodium* more accessible to the research community. Despite the absence of associated pathology, the liver stage of the malaria parasite life cycle acts as an important amplification step in the infection pathway and so has long been considered an attractive target for vaccine or drug-mediated prophylactic approaches to disease control. Our results show that inhibitors of SUB1 could be used in prophylactic approaches to control or block the pre-erythrocytic stage of the malaria parasite life cycle.

## Materials and Methods

### Ethics statement

Research using animals was approved by the Ethical Review Committee of the Wellcome Trust Sanger Institute and was conducted in accordance with the UK Animals (Scientific Procedures) Act 1986, under licence number PPL 80/2158 issued by the UK Home Office.

### Mice, mosquitoes and parasites

Tuck-Ordinary outbred mice and C57BL/6N inbred mice were used as 6–8 week old females. All animal procedures were carried out in accordance with a valid UK Home Office project licence. *Anopheles stephensi* strain SD500 mosquitoes were allowed to feed on mice 3–4 days after injection of infected blood, then maintained on fructose at 20°C. Sporozoite numbers were determined from day 21 by homogenising dissected salivary glands and counting released sporozoites using a haemocytometer. For conditional gene disruption experiments, mosquitoes were placed at 25°C from day 17–18 post infection and sporozoite isolation was performed from day 26 post infection. Parasite transfection experiments used the GFP-expressing *P. berghei* ANKA clone 507m6cl1 [40], [53] (kindly provided by Chris Janse and Shahid Khan, University of Leiden) for epitope-tagging of *pbsub1*, and the *P. berghei* NK65 UIS4-FlpL deleter clone [30] (a kind gift of Robert Menard, Institut Pasteur, Paris) for generation of the condSUB1 clones. The *P. berghei* NK65 UIS4-FlpL-F clone [41], which constitutively expresses GFP under the control of the *P. berghei hsp70* promoter, was used as a control for experiments with the condSUB1 clones; prior to isolation of control UIS4-FlpL-F sporozoites for hepatocyte infection experiments, mosquitoes infected with this line were also subjected to a temperature shift as described above for the condSUB1 parasites. Transfection of targeting constructs followed standard methodology [40]. Transgenic parasites were selected using pyrimethamine and cloned by limiting dilution.

### Antibodies, western blot and IFA

DNA encoding the predicted catalytic domain (residues Ser196-Asn599) of PbSUB1 (PBANKA_110710) was amplified by PCR using primers F_CatDPbSUB1synth_BamHI and R_CatDPbSUB1synth_XhoI (Table S1) and cloned into the bacterial expression vector pGEX-His (a kind gift of Dominique Soldati-Favre, University of Geneva, Switzerland) for expression as a recombinant hexahistidine-tagged protein in *E. coli* BL21-Gold DE3 cells (Stratagene). Recombinant product was purified by nickel chelate chromatography and used to immunise a rabbit. Before use, sera were adsorbed against *E. coli* acetone powder (40 mg/ml serum) to deplete antibodies against bacterial proteins. Other primary antibodies used were: the anti-HA.11 mouse mAb 16B12 (Covance); the anti-HA rat mAb 3F10 (Roche); the *P. berghei* MSP1-specific mAb 25.1 [54] (a gift from Tony Holder, NIMR, London, UK); a polyclonal antiserum against the PVM protein EXP1 [55], [56] (a kind gift from Volker Heussler, University of Bern, Switzerland); a polyclonal anti-HSP70 mouse antibody (a gift from Kai Matuschewski, Max Planck Institute, Berlin, Germany); a polyclonal antibody specific for PbSERA3, raised against a recombinant fragment of PbSERA3 called PbS3C1 [15]; and chicken and rabbit polyclonal anti-GFP sera (Abcam). Western blot analysis of *P. berghei* schizont SDS extracts and IFA analysis of paraformaldehyde-fixed, permeabilized blood stage parasites were performed as described previously [15], [57]. For IFA of parasitised hepatoma cells, infected cells were fixed for 15 min in 3% paraformaldehyde in phosphate-buffered saline (PBS), then quenched in 50 mM ammonium chloride in PBS. Samples were permeabilized with 0.1% (v/v) Triton X-100, washed, then incubated for 1 h in blocking solution (2% (w/v) BSA in PBS). Samples were probed with relevant primary antibodies diluted in blocking solution for 1 h, then with appropriate secondary antibodies before counterstaining with DAPI and observation using a laser scanning confocal microscope (LSM510, Zeiss). Primary antibodies were used for IFA at dilutions ranging from 1∶400–1∶1000. Secondary antibodies (usually used at a 1∶1000 dilution) were Alexa Fluor 488, 555 or 633-conjugated antibodies against mouse, rabbit or chicken IgG (Invitrogen).

### Generation of the pPbSUB1-HA construct for epitope tagging of the *pbsub1* gene

A 1,248 bp sequence corresponding to the 3′ coding sequence of the *pbsub1* gene was amplified from *P. berghei* gDNA using primers F_KI_XhoI and R_KI_ApaI (Table S1) and cloned into the transfection vector pSD278-HA (a kind gift of Dominique Soldati-Favre, University of Geneva, Switzerland), to produce an in-frame fusion to a single HA epitope tag, followed by the *pbdhfr-ts* 3′ UTR and a hDHFR drug selection cassette. Before transfection, the plasmid was linearised at a unique *Hind* III restriction site that lies 682 bp upstream of the stop codon of the *pbsub1* gene.

### Generation of the condSUB1 allelic exchange vector

To construct a conditional deletion vector for *pbsub1* we first identified a clone, PbG01-2474a09, from an arrayed and end-sequenced library of *P. berghei* ANKA cl15cy1 genomic DNA in *E. coli* [32] that carried *pbsub1* (PBANKA_110710) and flanking genes on a 9.6 kb insert in a low copy linear plasmid [58]. This clone served as starting point for engineering an allelic exchange vector using a combination of site specific and lambda phage recombinases in four steps (Figure S11 in Text S1), which used protocols essentially as described [59]. Each intermediate product was fully sequenced before moving on to the next stage. For Step 1 (Figure S11 in Text S1) we used lambda Red/ET mediated recombination with zeocin selection to insert a PCR amplicon ∼2.3 kb or ∼1.8 kb upstream of the *pbsub1* start codon that comprised an *hsp70* promoter followed by a *zeo-pheS* cassette for positive and negative selection in *E. coli*. The *zeo-pheS* cassette was flanked by two *FRT* sites in the same orientation and its insertion point was still 800 bp away from the start codon of the upstream gene. Primers used were F_Step1_rec and R_Step1_rec or F_Step1short_rec and R_Step1short_rec (Table S1) and plasmid pColE1 5′hsp70-ATG-FRT-zeo-pheS-FRT served as a template (see Supplemental Methods). Multiple stop codons were present downstream of the inserted sequence. In Step 2, the *FRT*ed *zeo-pheS* cassette was excised by inducing Flp-e recombinase expression in *E. coli* under negative selection against *pheS*, leaving behind only the *hsp70* promoter and one *FRT* site in the upstream intergenic region. In Step 3, Red/ET mediated recombination was used under zeocin selection to insert immediately upstream of the *pbsub1* stop codon a DNA fragment that introduced a single HA epitope tag followed by a generic 3′ UTR from the *pbdhfr-ts* gene as well as a *zeo-pheS* cassette flanked by *attR* recognition sites for Gateway clonase. The DNA fragment used in this step was release by a *Hind* III digest from plasmid pColE1 sub1-HA-attR1-zeo-pheS-attR2-3′sub1 (see Supplemental Methods). Finally, the product of Step 3 was subjected to an *in vitro* Gateway reaction under negative selection against *pheS*, which replaced the bacterial selection cassette with an *FRT* site immediately followed by a GFP coding sequence, an *hsp70* terminator sequence and an expression cassette for *hdhfr-yfcu* for selection in *P. berghei*. In the final construct, *FRT* sites were positioned such that excision of *pbsub1* would bring an ATG start codon 5′ of the upstream *FRT* site in frame with the *gfp* coding sequence, allowing expression of the fluorescent marker protein from the *hsp70* promoter. The construct was tested by Flp-e activation in *E. coli* TSA cells, followed by PCR genotyping of the excised vector. See Supplemental Methods for more details on vector generation.

### Genotyping PCR and Southern blot analysis

Genomic DNA was extracted from blood stage parasites, infected mosquito midguts or infected salivary glands. For Southern blot analysis, the DNA was digested with suitable restriction enzymes and separated by gel electrophoresis. Transfer of the DNA to a nitrocellulose membrane and hybridisation with gene-specific probes was performed according to standard procedures. Probes were labelled with α-[^32^P] adenosine triphosphate (Amersham Biosciences) by random priming using a Prime-It Random Prime kit (Stratagene).

To detect integration of the transfected pPbSUB1-HA construct, primers Fprom_PbSUB1, F2_PbSUB1and R_HA were used. The size of the expected PCR products - which could only be produced if integration occurred as predicted - were 1,942 bp and 1,288 bp respectively. Two other control PCRs were generated using primers F1_PbSUB1, Fprom_PbSUB1, R1_3′utr. These were expected to produce 1,937 bp and 2,036 bp products only from the unmodified wild type *pbsub1* locus. For Southern blot analysis, parasite genomic DNA was digested with *Pml* I and *Nhe* I. An 841 bp probe annealing to the 5′ flanking sequence of *pbsub1* was generated with primers F_prom_probe and R_prom_probe. This allowed detection of a 4.6 kb band for the wild type *pbsub1* locus and a 10.6 kb band for the expected integration event.

To detect integration of the pJazz-FRTed-pbsub1 constructs, primer F_selection (forward) was used with reverse primers R_ext1, R_ext2, or R_ext3 to produce PCR products of 1,471 bp, 2,034 bp and 2,617 bp respectively. As control PCRs for detection of the unmodified *pbsub1* locus primers F2_PbSUB1 or F3_PbSUB1 were used together with R1_3′utr. These were predicted to produce products of 1,382 bp and 598 bp only from the unmodified *pbsub1* locus. For Southern blot analysis, parasite genomic DNA was digested using *Stu*I and *Nci*I, and a 1,246 bp probe annealing to the *pbsub1* gene was generated with primers F_KI_XhoI and F_KI_ApaI. This allowed detection of a 3 kb band for the unmodified *pbsub1* locus and an 8.4 kb band for *P. berghei* condSUB1 and condSUB1_short_ parasites.

Intact *P. berghei* chromosomes were separated by pulsed-field gel electrophoresis on 0.8% agarose gels as described previously [32]. Gels were blotted and hybridised using the standard Southern blot protocol. For detection of integration in the correct chromosome a 452 bp probe hybridising to the *pbdhfr-ts* 3′ UTR was generated with primers F_3′utr_pbdhfr_probe and R_3′utr_pbdhfr_probe (Table S1).

### *In vitro* hepatoma cell infection and merosome release assays

Hepa1-6 mouse or HepG2 human hepatoma cells were cultured in Dulbecco's Modified Eagle Medium high glucose (DMEM; Gibco/Invitrogen) plus 10% (v/v) fetal calf serum (Invitrogen). For sporozoite infection rate assays and determination of parasite size at 28 and 48 h post infection, cells were seeded in 96-well culture plates (10,000–15,000 cells per well). The next day, 20,000 or 50,000 control or condSUB1 sporozoites were added to each well in complete medium containing 1% penicillin/streptomycin. Following incubation for 2–48 h, cells were washed four times with PBS then fixed and processed for IFA. For differential extracellular/intracellular staining (in/out assay), staining was performed as described previously [44] using a Cy3 conjugated mouse anti-CS antibody, a rabbit Alexa Fluor 488 anti-GFP antibody (Invitrogen) and Hoechst 33342 (Molecular Probes/Invitrogen).

For the in/out assay and the determination of parasite area at 28 h and 48 h post invasion, images of fixed, stained wells were automatically acquired with a Cellomics ArrayScan VTI HCS Reader (20× magnification; 100 or 155 fields per well of a 96-well plate) and analysed using the Colocalization BioApplication software (ThermoFisher). For the in/out assay, object recognition was based on the GFP staining and average intensity of the CSP-specific signal was used to distinguish extracellular (high) and intracellular (low) sporozoites. The number of cell nuclei was determined based on the Hoechst signal and used to calculate invasion rates. For the parasite size assay, number of cell nuclei and number and area of parasites were determined. Object recognition was based on the HSP70-specific staining, applying a minimum object size of 15 µm^2^ at 28 h post invasion and 50 µm^2^ at 48 h post invasion. The average intensity of the GFP staining was used to discriminate excised and non-excised condSUB1 parasites. The cell nuclei were counted with help of the Hoechst signal and numbers used to calculate infection rates. For each comparison, Student's *t*-test was performed and for the invasion rates the threshold of the p value was set to 0.05, whereas for parasite sizes a Bonferroni corrected threshold of the p value was chosen at 0.0167 (*) or 0.0033 (**).

To assess release of merosomes and detached cells in hepatoma cultures, 40,000–80,000 cells were seeded into 24-well plates one day before sporozoites were added at a multiplicity of infection of 1. Merosomes were collected from the culture supernatants between 65 and 69 h post infection, fixed in suspension, pelleted, resuspended in a small volume of medium, fixed onto poly-L-lysine coated microscope slides (Menzel-Gläser), and stained with anti-GFP, anti-MSP1, anti-EXP1 antibodies and DAPI before analysis by fluorescence microscopy.

### Parasite liver load assays

C57BL/6N mice were injected intravenously with 20,000 *P. berghei* sporozoites and livers were collected at 40 h post infection. Livers were mechanically homogenized on ice with a Tissue Tearor (IKA Ultra Turrax T-10) in 4 ml denaturing solution (4 M guanidine thiocyanate, 25 mM sodium citrate pH 7, 0.5% N-Laurosyl-sarcosine, 0.1% β-mercaptoethanol) total RNA extracted using an RNeasy Mini Kit (Qiagen). Samples were treated with Turbo DNAse (Ambion) according to the manufacturer's instructions. One microgram of total RNA was reverse-transcribed using a Transcriptor First Strand cDNA Synthesis kit (Roche). Parasite 18S ribosomal RNA and mouse hypoxanthine guanine phosphoribosyltransferase (HPRT) cDNAs obtained from the reaction were quantified by real-time quantitative fluorogenic PCR using previously described primers respectively F_Pb18S and R_Pb18S for *P. berghei* 18S ribosomal RNA, and F_HPRT and R_HPRT for the *Mus musculus* housekeeping gene HPRT gene. To quantify gene expression, Power SYBR Green PCR Master Mix (Applied Biosystems) was used according to the manufacturer's instructions. The reaction was performed in an ABI Prism 7000 sequence Detection System (Applied Biosystems) with 2 µl of cDNA in a total volume of 25 µl and the following reaction conditions: 1 cycle of 2 min at 50°C, 1 cycle of 10 min at 95°C, 50 cycles of 15 sec at 95°C and 1 min at 60°C. Each sample was assayed in triplicate. Relative amounts of RNA were calculated using the ABI Prism 7000 SDS 1.2.3 Software, and normalised against expression levels of the mouse HPRT mRNA.

## Supporting Information

Text S1 Supplemental Figures S1 to S11, Supplemental Methods, and Table S1.
(PDF)
