# Supplementary material for: The Malarial Serine Protease SUB1 Plays an Essential Role in Parasite Liver Stage Development
Source: PLoS Pathog. 2013 Dec 12;9(12):e1003811. doi: 10.1371/journal.ppat.1003811 (PMC3861531; doi:10.1371/journal.ppat.1003811)
Supplement: Text S1 — Supplemental Figures S1 to S11, Supplemental Methods, and Table S1. (PDF) [file ppat.1003811.s001.pdf]

Supplemental Data and Methods

The malarial serine protease SUB1 plays an essential role in parasite liver stage development

Catherine Suarez[1], Katrin Volkmann[2], Ana Rita Gomes[2], Oliver Billker[2*] and Michael J. Blackman[1*]

[1]Division of Parasitology, MRC National Institute for Medical Research, Mill Hill, London NW7 1AA, UK

[2] Wellcome Trust Sanger Institute, Hinxton, Cambridge CB10 1SA, UK

[*]Correspondence: ob4@sanger.ac.uk or mblackm@nimr.mrc.ac.uk. Tel. +44 208 816 2127. Fax.: +44 208 816 2730

Running title: PbSUB1 is essential in malarial liver stages

Figure S1. Detection of PbSUB1 in *P. berghei* blood and liver stage schizonts using an anti-PbSUB1 antibody. (A) SDS extracts of *P. berghei* ANKA schizonts were probed with a rabbit antibody raised against the putative catalytic domain (residues Ser196-Asn599) of PbSUB1 (PBANKA_110710). By analogy with the known proteolytic maturation profile of parasite-derived *P. falciparum* SUB1 [1,2], as well as the processing pattern observed with recombinant SUB1 from *P. falciparum*, *P. vivax*, *P. knowlesi* and *P. berghei* [3,4], the signal at ~70 kDa likely corresponds to full-length unprocessed PbSUB1 zymogen, which includes its N-terminal prodomain. *P. falciparum* SUB1 undergoes autocatalytic maturation in two steps, involving first loss of the prodomain then a further smaller N-terminal truncation to form a terminal product comprising predominantly the catalytic domain [1,2]. The doublet band at 44-47 kDa therefore likely corresponds to the two processed forms of PbSUB1. (B) Detection of PbSUB1 in infected hepatoma cells. Hepa1-6 cells infected with sporozoites of the marker-free, GFP-expressing *P. berghei* 507m6cl1 (RMgm-7) clone [5] (also see the RMgm database at http://www.pberghei.eu/index.php), were fixed 64 h post infection then probed with the rabbit anti-PbSUB1 antibodies (red), anti-GFP antibodies (green), and the DNA dye 4,6-diamidino-2-phenylindol (DAPI; white or blue), and examined by confocal microscopy. The extreme right-hand image is enlarged in order to better visualize the relative localization of the PbSUB1-specific signal relative to the parasite nuclei. TL-transmitted light. Scale bar, 10 µm.

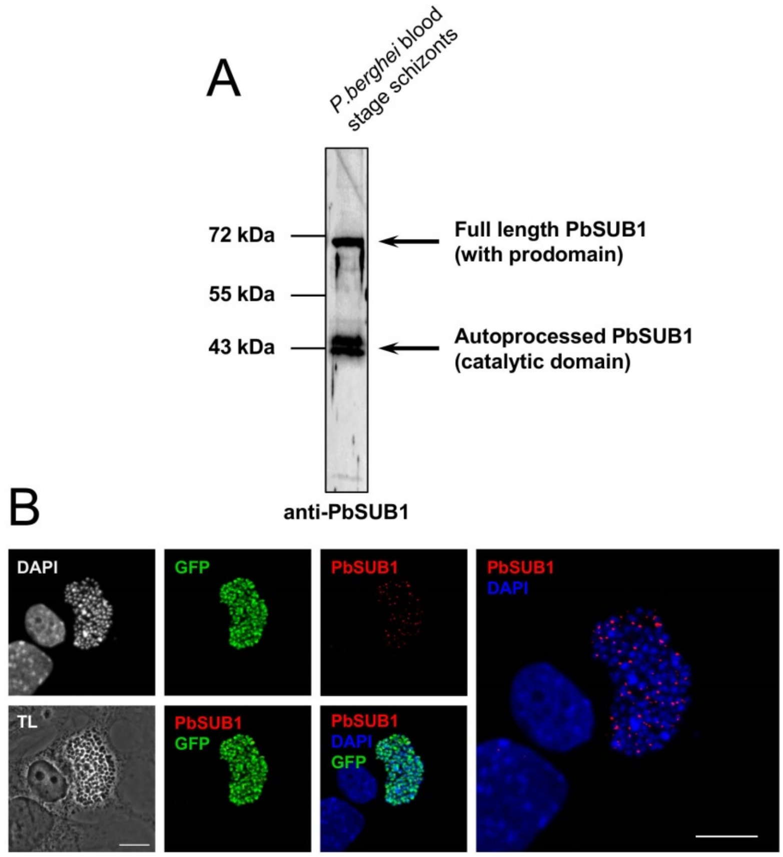

Figure S2. Epitope tagging of the endogenous *pbsub1* gene. (A) Schematic outlining the single-crossover homologous recombination strategy used to fuse a single hemagglutinin (HA) epitope tag (red) to the *pbsub1* coding sequence (orange). The targeting plasmid, called pPbSUB1-HA, contained 1,248 bp of targeting sequence (hatched pale orange) fused to the tag, followed by the 3' UTR sequence from the *P. berghei* dihydrofolate reductase thymidylate synthase (*pbdhfr-ts*) gene to ensure correct transcription termination and polyadenylation of the modified gene. The presence of the human dihydrofolate reductase (hDHFR) cassette allowed selection of integrant parasites with pyrimethamine. The integration construct was linearised by restriction digestion at a unique *Hind* III site prior to transfection into the GFP-expressing *P. berghei* 507m6cl1 (RMgm-7) clone [5]. Positions of hybridization of primers used for diagnostic PCR analysis of the wild-type and modified loci are indicated (red arrows), as well as the predicted size of PCR amplicons (red dotted lines). Primers used were Fprom_PbSUB1 (a), R_HA (b), F2_PbSUB1 (c), F1_PbSUB1 (d) and R1_3'utr (e) (sequences of all oligonucleotide primers used in this study are provided in

Supplemental Table S1 at the end of this file). (B) Southern hybridisation of pulse field gel-separated chromosomes of cloned PbSUB1-HA and control non-transfected parental parasites confirmed integration of the tagging construct into the expected chromosome 11 location. For detection, a 452 bp probe hybridising to the *pbdhfr-ts* 3' UTR was generated with primers F_3'utr_pbdhfr_probe and R_3'utr_pbdhfr_probe (Table S1). Note that the signal at the position of chromosome 7 observed in both the parental and PbSUB1-HA tracks corresponds to the endogenous *pbdhfr-ts* gene. The stronger signal observed at chromosome 11 in the PbSUB1-HA lane is due to hybridisation of the probe to both the modified *pbsub1* gene as well as the integrated hDHFR cassette, which also contains the *pbdhfr-ts* 3' UTR (not shown).

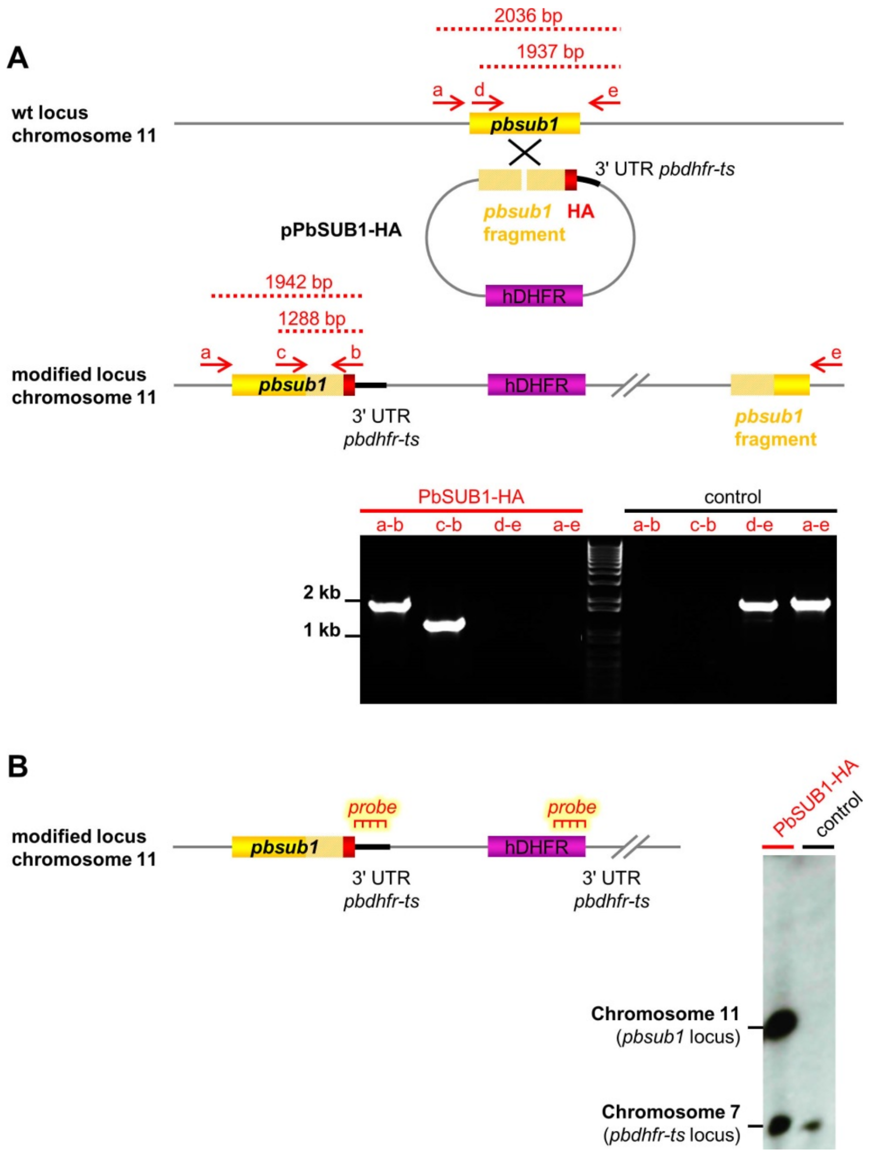

Figure S3. Expression of epitope-tagged PbSUB1 in asexual blood stage schizonts but not in salivary gland sporozoites. (A) Western blot of extracts from PbSUB1-HA blood stage schizonts and parental control *P. berghei* clone 507m6cl1, probed with either rabbit anti-PbSUB1 antibodies or an anti-HA monoclonal antibody (mAb). The signals obtained likely correspond predominantly to the processed (mature) forms of PbSUB1 with only very faint signals for the full-length unprocessed proteins, as usually observed in the case of *P. falciparum* SUB1 [2,6]. (B) Immunofluorescence analysis (IFA) of fixed blood stage schizonts of the PbSUB1-HA and parental clones, probed with anti-HA and anti-GFP antibodies. (C) IFA of fixed PbSUB1-HA salivary gland sporozoites, probed with anti-HA, anti-GFP and anti-UIS4 antibodies. No HA signal was detectable in the sporozoites. Scale bars, 5 µm.

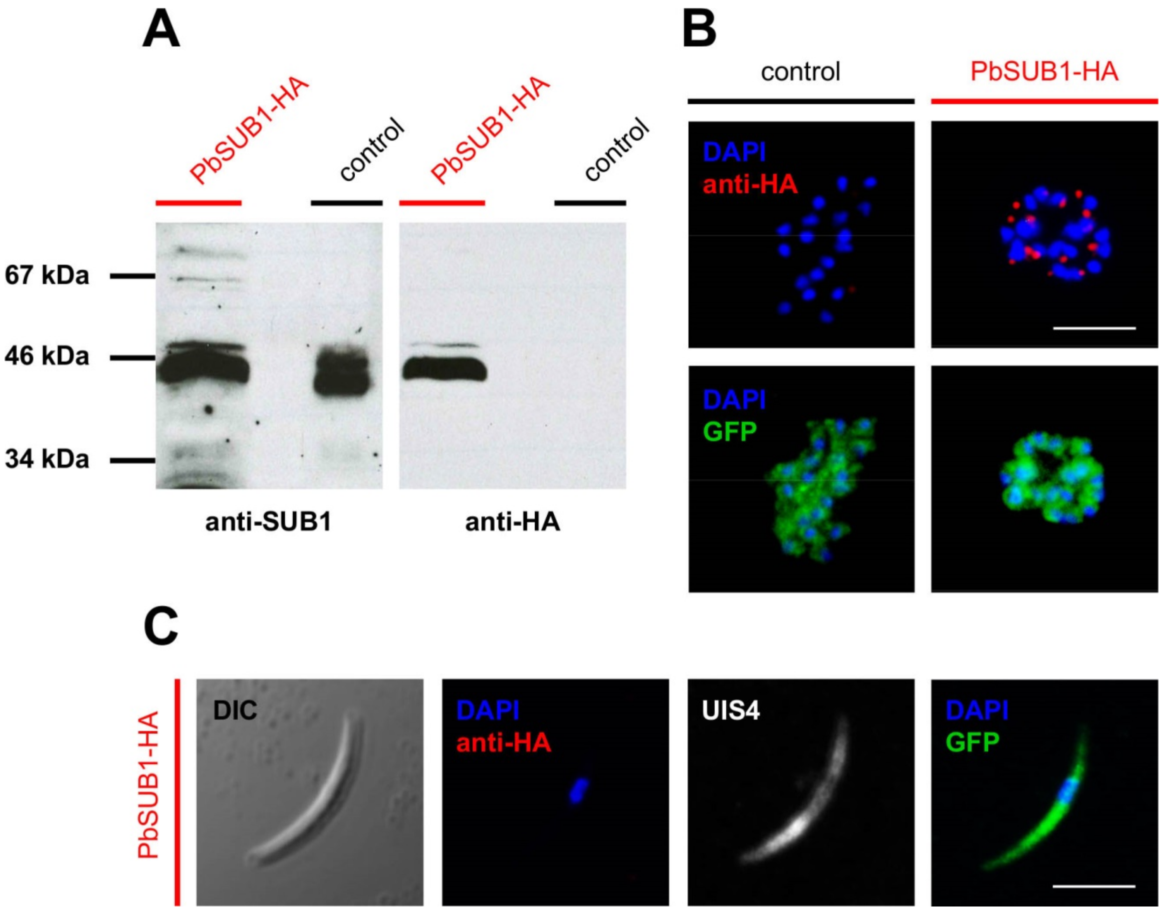

Figure S4. Detection of epitope-tagged PbSUB1 in maturing but not early liver stage schizonts. HepG2 hepatoma cells infected *in vitro* with sporozoites of the PbSUB1-HA clone or parental GFP-expressing *P. berghei* clone (control) were fixed and examined by IFA, probing with anti-HA or anti-GFP antibodies. Note the lack of signal in the early PbSUB1-HA schizonts (top row), in which formation of individual merozoites is not yet visible. Scale bar, 10 µm.

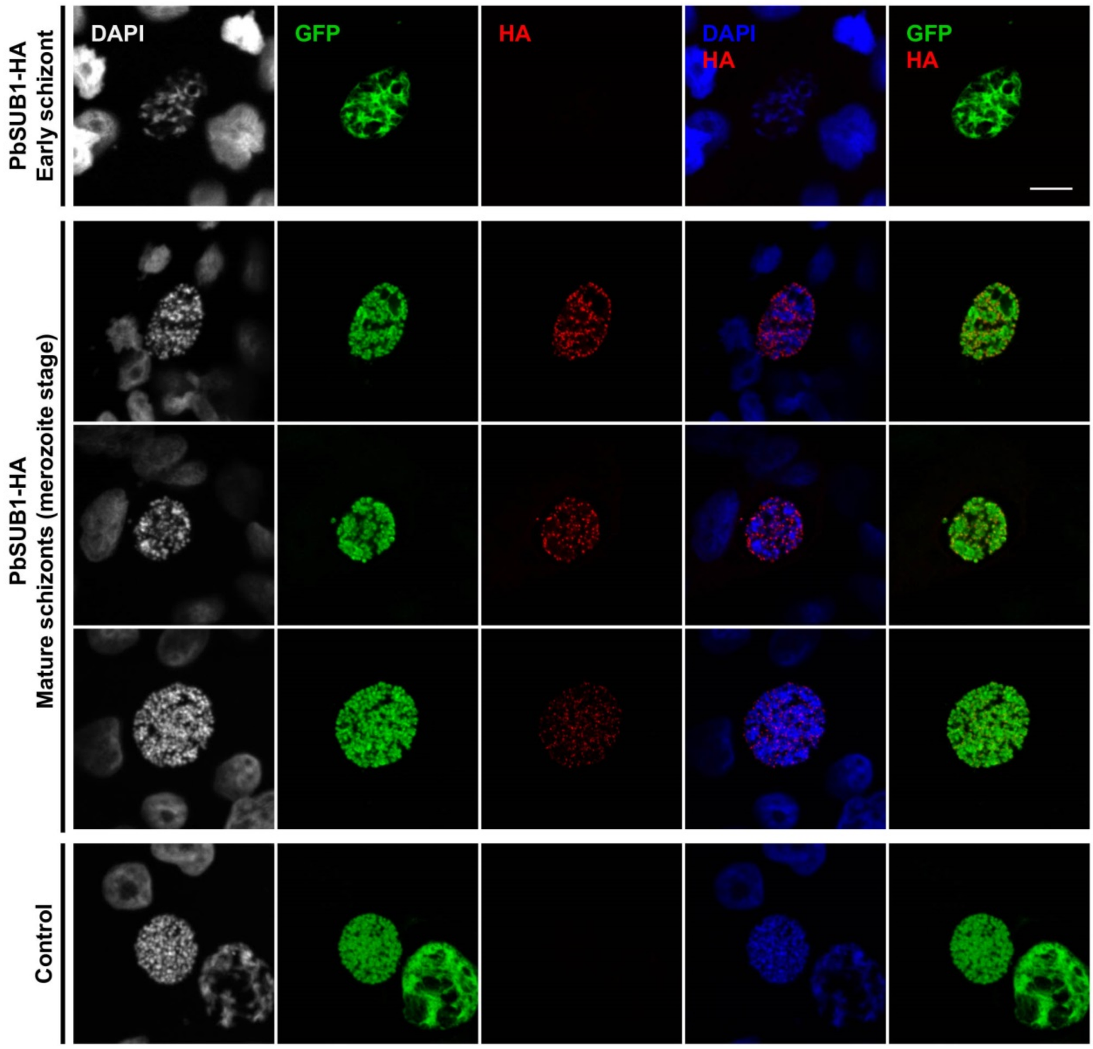

Figure S5. Epitope-tagged PbSUB1 is detectable in mature liver stage schizonts but not at the cytomere stage. HepG2 hepatoma cells infected *in vitro* with sporozoites of the PbSUB1-HA clone were fixed and examined by IFA, probing with anti-GFP, anti-HA, or anti-MSP1 antibodies. Note the lack of HA signal in the cytomere stage schizont, characterised by the absence of individual merozoites but the presence of an invaginated schizont plasma membrane (detected by the anti-MSP1 antibodies) surrounding groups of parasite nuclei. Scale bar, 10 µm.

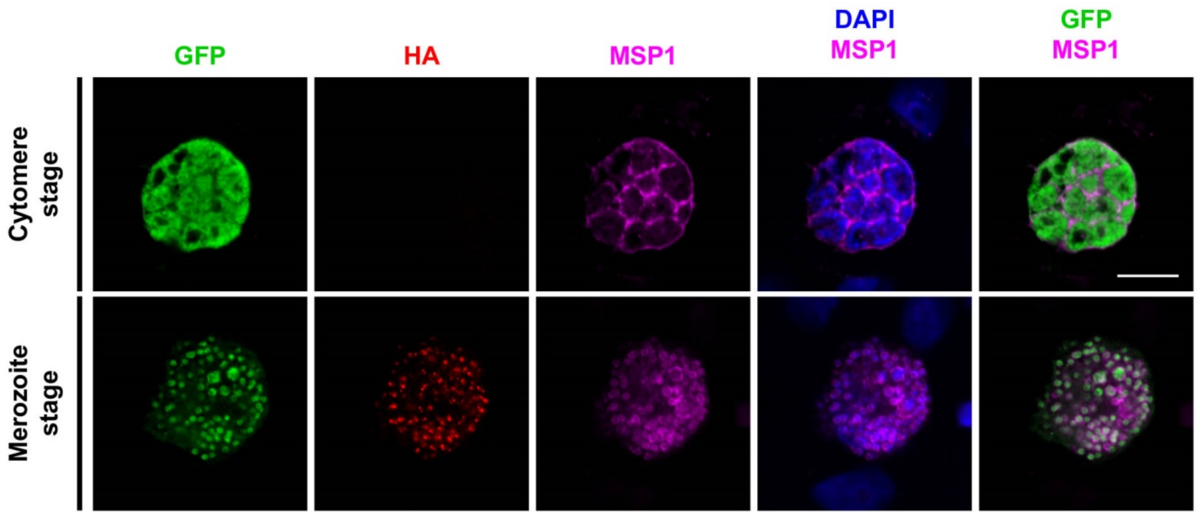

Figure S6. Allelic exchange of wild type *pbsub1* with a conditional allele for Flp mediated deletion by ends-out recombination. (A) Genotyping PCR confirms the genomic structure of the modified *pbsub1* locus in the blood stage condSUB1 and condSUB1$_{short}$ parasite clones recovered following transfection with the pJazz-FRTed-pbsub1 constructs (see Figure 2A of main manuscript and Figure S7 below for a full description of the constructs). Shown is a schematic of the predicted modified *pbsub1* locus, as well as the wild type locus, and the positions of hybridisation of a range of PCR primers (red arrows) as well as the predicted sizes of resulting amplicons (red dotted lines). PCR reactions a-b, a-c and a-d are expected to produce products only with the modified (integrated) locus, whilst PCR reactions f-g and e-g are specific for the unmodified locus.

Primer identities are: F_selection (a); R_ext1 (b); R_ext2 (c); R_ext3 (d); F2_PbSUB1 (e); F3_PbSUB1 (f); and R1_3'utr (g) (see Table S1). (B) Southern blot. Asexual blood stage genomic DNA of condSUB1$_{short}$, condSUB1 and parental control parasites was digested with *Stu* I and *Nci* I and hybridised with a 1.3 kb probe corresponding to an internal segment of *pbsub1*. (C) Southern hybridisation on pulse field gel-separated chromosomes, using a 452 bp probe corresponding to the *pbdhfr-ts* 3' UTR. This sequence is present in the endogenous *pbdhfr-ts* locus as well as in the modified *pbsub1* locus, as it is used as a 3' UTR for the flirted *pbsub1* gene in all the transgenic parasites. Data are shown only for condSUB1 clone A and the condSUB1$_{short}$ clone, but were identical for condSUB1 clone B.

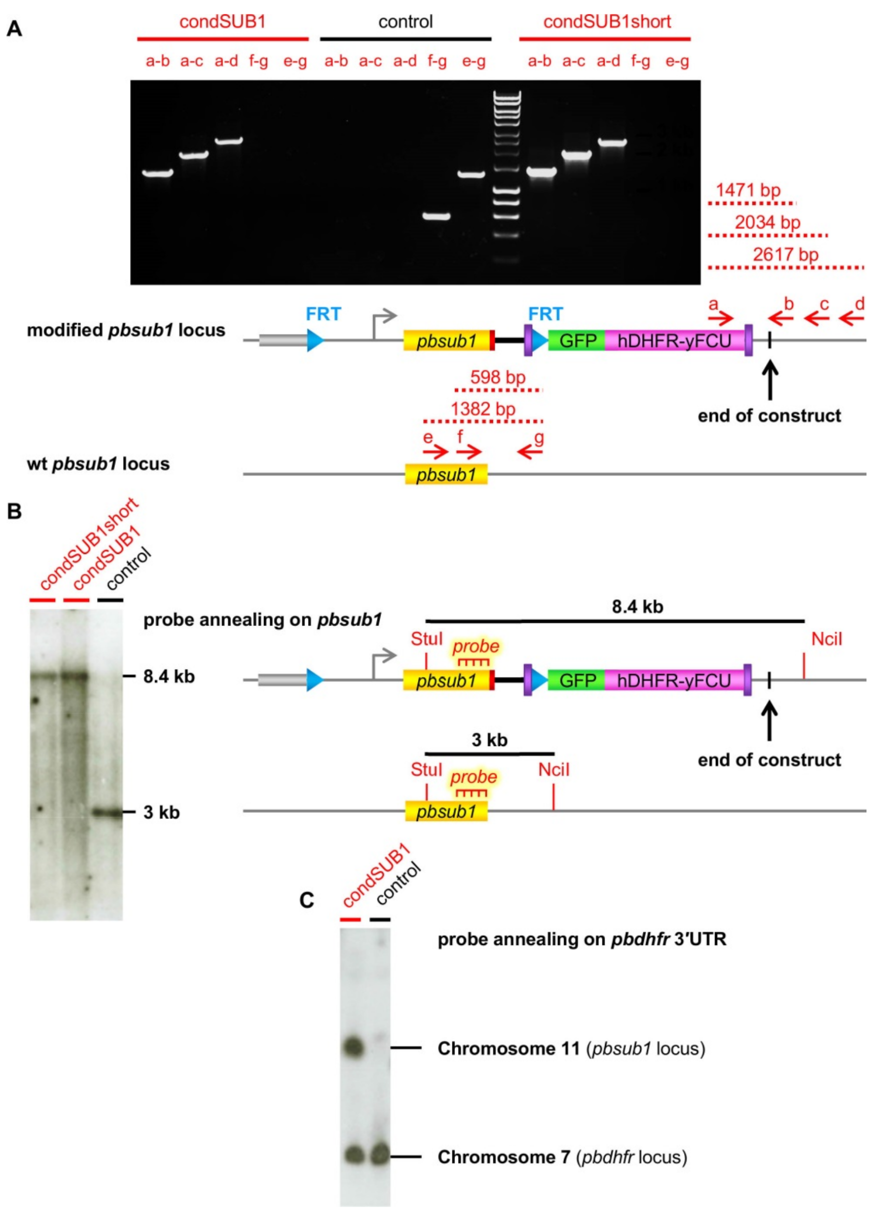

Figure S7. Insect stage-specific conditional deletion of the *pbsub1* gene blocks the transition from salivary gland sporozoite to subsequent asexual blood stages. (A) Double crossover homologous recombination strategy to simultaneously flirt and epitope-tag the *pbsub1* gene using the pJazz-FRTed-pbsub1$_{short}$ construct. A ~10 kb genomic DNA library clone containing the *pbsub1* gene was modified by recombineering and Gateway technology to place an *FRT* site ~1.8 kb upstream of the *pbsub1* gene and directly downstream of an inserted *P. berghei hsp70* promoter (this differs from the placement of the *FRT* site and inserted *P. berghei hsp70* promoter in the pJazz-FRTed-pbsub1 construct; see Figure 2 in the main paper). A second downstream *FRT* site was inserted in frame with a GFP reporter coding sequence so that, upon excision, the *hsp70* promoter drives expression of GFP. The final construct was transfected into the *P. berghei* UIS4/FlpL clone and an integrant parasite clone obtained, called condSUB1$_{short}$. (B) Mosquitoes fed on donor mice infected with condSUB1$_{short}$ parasites were subjected to a temperature shift 18 days post transmission to ensure optimal activity of the FlpL recombinase. Oocysts, salivary glands and sporozoites from these insects displayed strong GFP expression at 26 days (d26), when the insects were allowed to feed on naive mice (bite-back). The resulting blood stage parasites were collected and analysed by PCR using the indicated primer pairs, which were expected to produce a ~1.2 kb amplicon from the non-excised modified *pbsub1* locus, or a ~300 bp or a ~600 bp product from the excised locus. Primers used were: (a) F3_PbSUB1 with R_out GFP (product 1203 bp; 'non-excised-specific' band); (b) F_out_hsp70 with R2_out GFP (product 281 bp; 'excised-specific' band); and (c) F_out_hsp70 with R_out GFP (product 605 bp, 'excised-specific' band) (see Table S1 for primer sequences). Results are also shown from analysis of the condSUB1 clone B (right hand side gel). Excision occurred efficiently in the d26 sporozoites, though in this case the non-excised locus was still detectable by PCR. However, as with the condSUB1 clone A (see Figure 2 of the main paper), only residual non-excised parasites were capable of establishing a blood stage infection in the bite-back mice. Microscopic examination of dissected d26 condSUB1$_{short}$

sporozoites showed that the proportion of GFP-positive sporozoites was generally ~90% (data not shown).

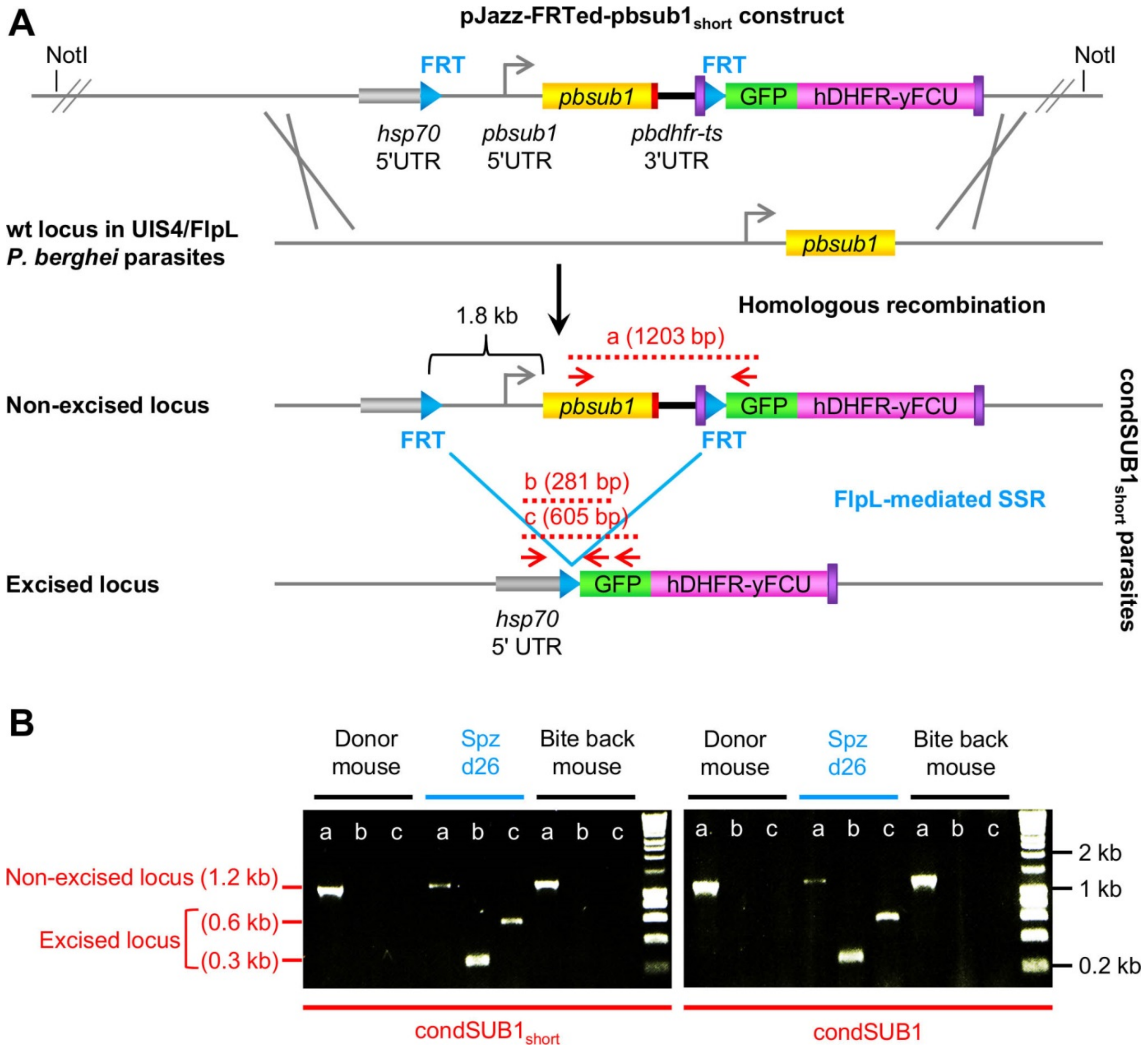

Figure S8. PbSUB1-deficient EEFs display a normal expression profile of the PV marker PbSERA3 and the PVM marker EXP1 during early schizont development. HepG2 cells infected with sporozoites of the indicated clones were fixed 48-56 h post infection and probed with antibodies against PbSERA3 (red), EXP1 (magenta), or GFP (green). DAPI-stained nucleic acids are shown in white or blue. Scale bar, 10 µm.

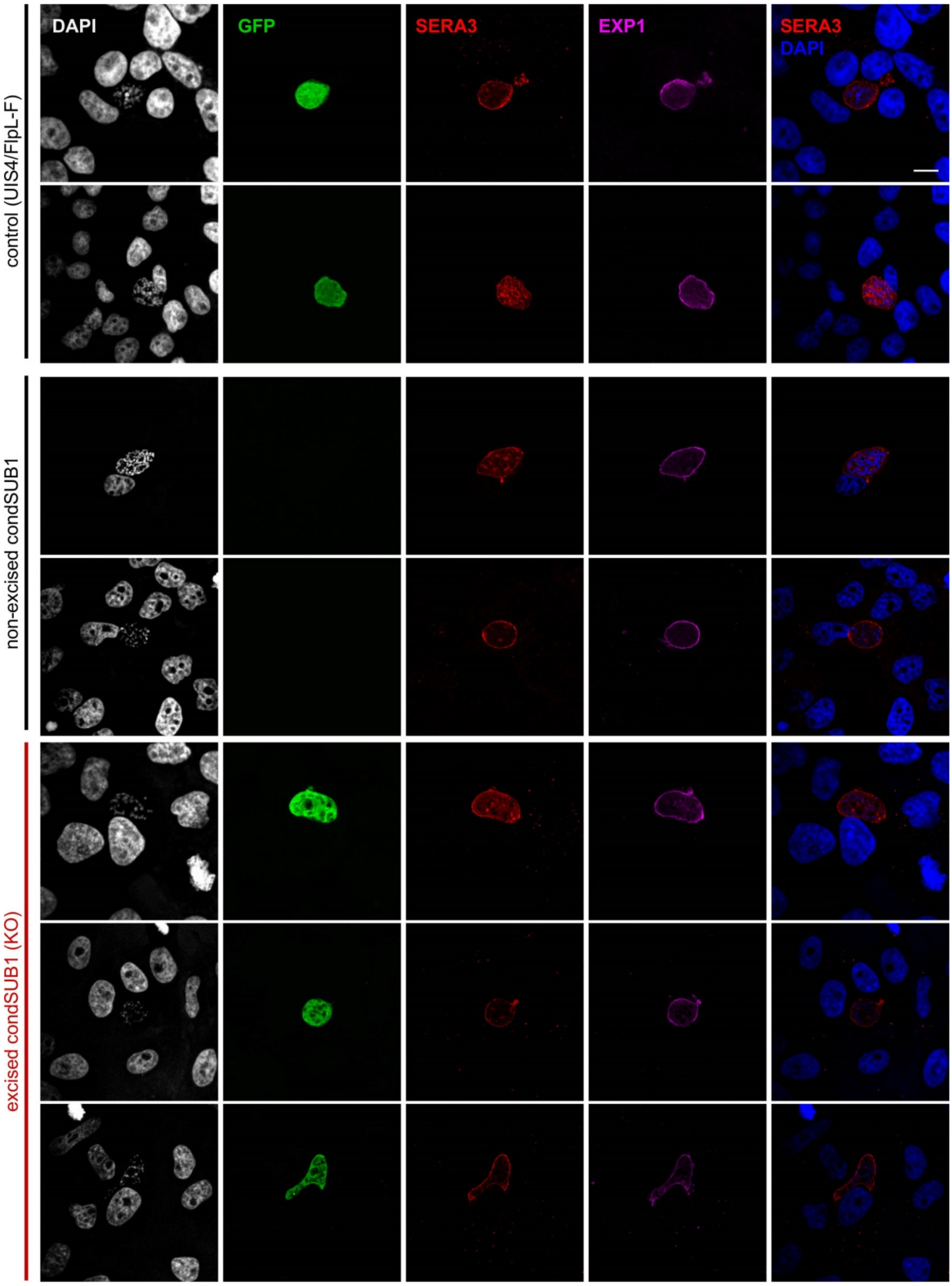

Figure S9. IFA analysis of merosomes. Representative GFP-expressing and non-GFP expressing merosomes released into the medium of control UIS/FlpL-F and condSUB1-infected HepG2 cells respectively, between 62 and 70 h post invasion. The dispersed signal obtained with antibodies to the PVM marker EXP1 is consistent with the presence of a ruptured PVM. The host cell nucleus is present in all merosomes depicted. Scale bar, 10 µm. BF, bright field.

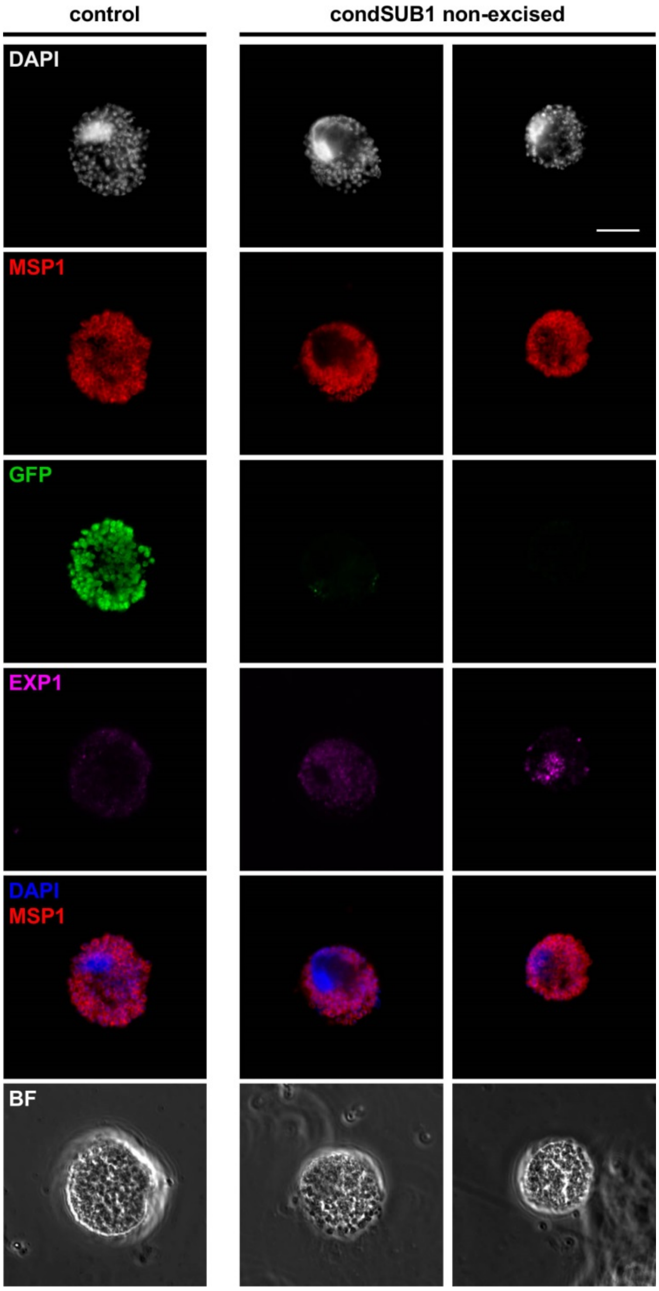

Figure S10. PbSUB1 is essential for production of merosomes. Supernatants harvested from equal numbers of HepG2 cells 62-70 h following infection with condSUB1 (~80% excised) or control UIS4/FlpL-F sporozoites (multiplicity of infection = 1) were centrifuged to pellet detached cells and merosomes. These were fixed onto poly-L lysine coated slides, stained for IFA using antibodies against MSP1 and GFP, then counted microscopically. Raw counts are shown. Each value shown was obtained from a total of 15 wells containing ~60,000 HepG2 cells/well, pooled from a total of 6 individual experiments.

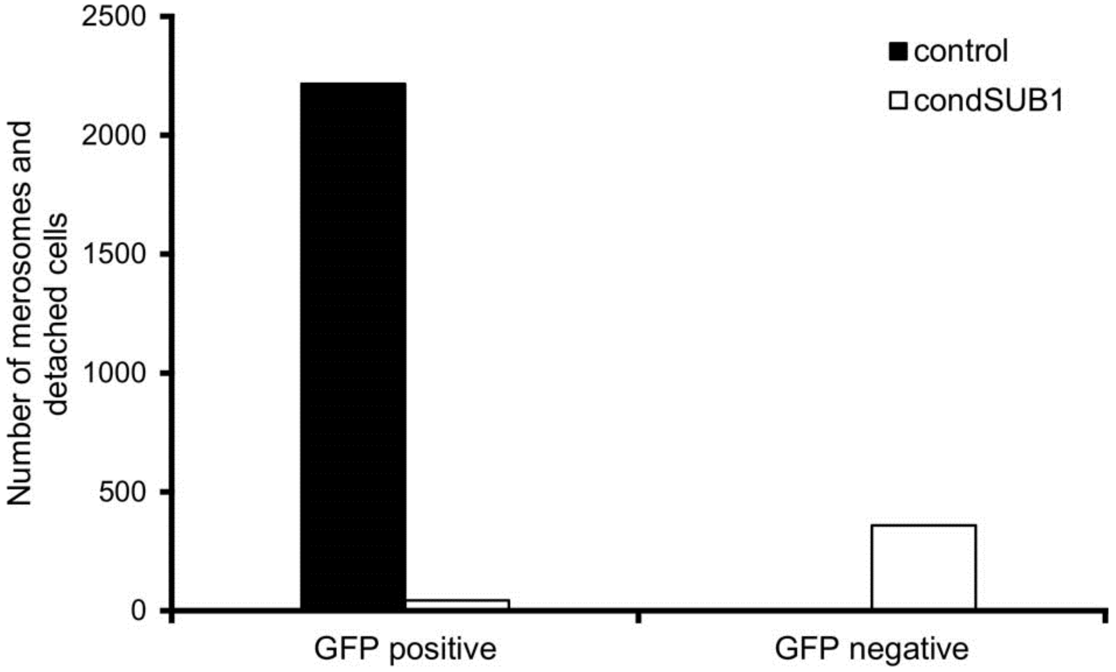

Supplemental Methods

Generation of recombineering tools for modification of the PbSUB1 locus

PCR template pColE1 5'hsp70-ATG-FRT-zeo-pheS-FRT for Step 1

The plasmid p5'hsp70-GFP (a kind gift from R. Menard, Institut Pasteur, Paris) containing GFP under the control of 5' and 3' regulatory sequences from *P. berghei hsp70* [7] was linearised immediately downstream of the 5' UTR with *Nhe* I. A PCR product on the *zeo-pheS* bacterial selection cassette of plasmid pR6K attR1-zeo-pheS-attR2 was generated using oligonucleotides F_FRT_Zeo_infu_pStep1 and R_FRT_Zeo_infu_pStep1 (Table S1), which contained 5' extensions to introduce *FRT* sites on either end, followed by 15 bp sequences for in-fusion cloning, and inserted into the linearised p5'hsp70-GFP plasmid.

DNA fragment sub1-HA-attR1-zeo-pheS-attR2-3'sub1 for Step 3

To create a DNA fragment for modifying the 3' end of *pbsub1* in the gDNA library clone (Step 3) by Red/ET mediated recombination we placed a bacterial cassette for positive and negative selection in *E. coli* (*zeo-pheS*) next to the 3' end of the *pbdhfr-ts* derived 3' UTR within the HA-tagging vector for *pbsub1*. For this a PCR amplicon that also retained the *attR* sites flanking *zeo-pheS* was generated from plasmid pR6K attR1-zeo-pheS-attR2 using the oligonucleotides F_Zeo_infu_pStep3 and R_Zeo_infu_pStep3 (Table S1). The reverse primer encoded a 5' extension corresponding to 54 bp homologous to the *pbsub1* 3' UTR, which were followed by a *Hin*d III site and 15 bp extensions for in-fusion cloning into plasmid pPbSUB1-HA, which had been linearised downstream of the *pbdhfr-ts* 3' UTR with *Bst* BI. Digestion of the resulting plasmid with *Hin*d III released a DNA fragment flanked by sequences from the *pbsub1* locus that was gel purified and used for Red/ET mediated homologous recombination in *E. coli* in Step 3.

Gateway donor pR6K attL1-FRT-GFP-hdhfr-yfcu-attL2 for Step 4.

We generated a gateway donor cassette to serve as a universal tool for introducing FRT sites downstream of genes. Using in-fusion cloning we assembled three DNA fragments in a plasmid backbone with a tetracycline resistance cassette and an R6K origin, replication of which is restricted to *pir*$^+$ strains of *E. coli*, thus preventing replication in *E. coli* TSA, which we used to propagate the product of the Gateway reaction in Step 4. Between the appropriate *attL* clonase recognition sites we assembled a promoterless GFP with a terminator from *hsp70*, which was followed in the same orientation by an expression cassette for *hdhfr-yfcu* for positive and negative selection in *P. berghei*. The *P. berghei* marker contained a promoter from *eef1αa* and a terminator from *hsp70*. The directly repeated 3' UTR from *hsp70* was designed to allow marker recycling in *P. berghei* by negative selection against *yfcu*. The resulting plasmid, pR6K attL1-FRT-GFP-hdhfr-yfcu-attL2, was verified by sequencing.

Red/ET mediated genetic engineering

All *in vivo* recombination reactions were carried out in 4 ml cultures following the protocol of Pfander et al. [8]. *E. coli* TSA harbouring the gDNA library clone PbG01-2474a09 that contains the *pbsub1* gene, were first electroporated with the recombinase plasmid pSC101gbaA-tet and the culture was grown overnight at 30˚C. The next day, the culture was diluted in fresh medium to an optical density (OD_{600}) = 0.05. When the OD_{600} reached 0.3 - 0.4, expression of the recombinase operon proteins was induced by addition of L-arabinose (0.2% w/v final) into the culture medium and the temperature was shifted to 37˚C. This allows efficient expression of the recombineering proteins and concomitant loss of the plasmid, which contains a thermosensitive pSC101 origin of replication. After 40 min of induction, the cells were washed with ice-cold ultrapure water and electroporated with the desired PCR product containing on either side a minimum of 40 bp homologous to the target

site. Cultures were allowed 70 min to recover before being spread onto plates containing appropriate antibiotics.

Flp recombinase reactions

The plasmid encoding the enhanced Flp recombinase, pSC101 708-Flp-e cm^R (Gene Bridges) was used in a similar way to the recombineering plasmid. To induce Flp-e expression and concomitant loss of the plasmid, the temperature was shifted to 37˚C when the culture was in log phase. After induction overnight, the 'flipped' clones were selected on YEG-Cl plates containing p-chlorophenylalanine to select for loss of the *zeo-pheS* cassette. After each recombineering and flipping step, the resulting clones were genotyped by PCR and confirmed by nucleotide sequencing. After an overnight culture, miniprep DNA was extracted before being extensively diluted and re-electroporated into TSA cells to eliminate carry-over of undesirable products that might interfere with the next modification step.

Figure S11. Generation of recombineering tools for modification of the PbSUB1 locus.

See Supplemental Methods text for a full explanation of the process used.

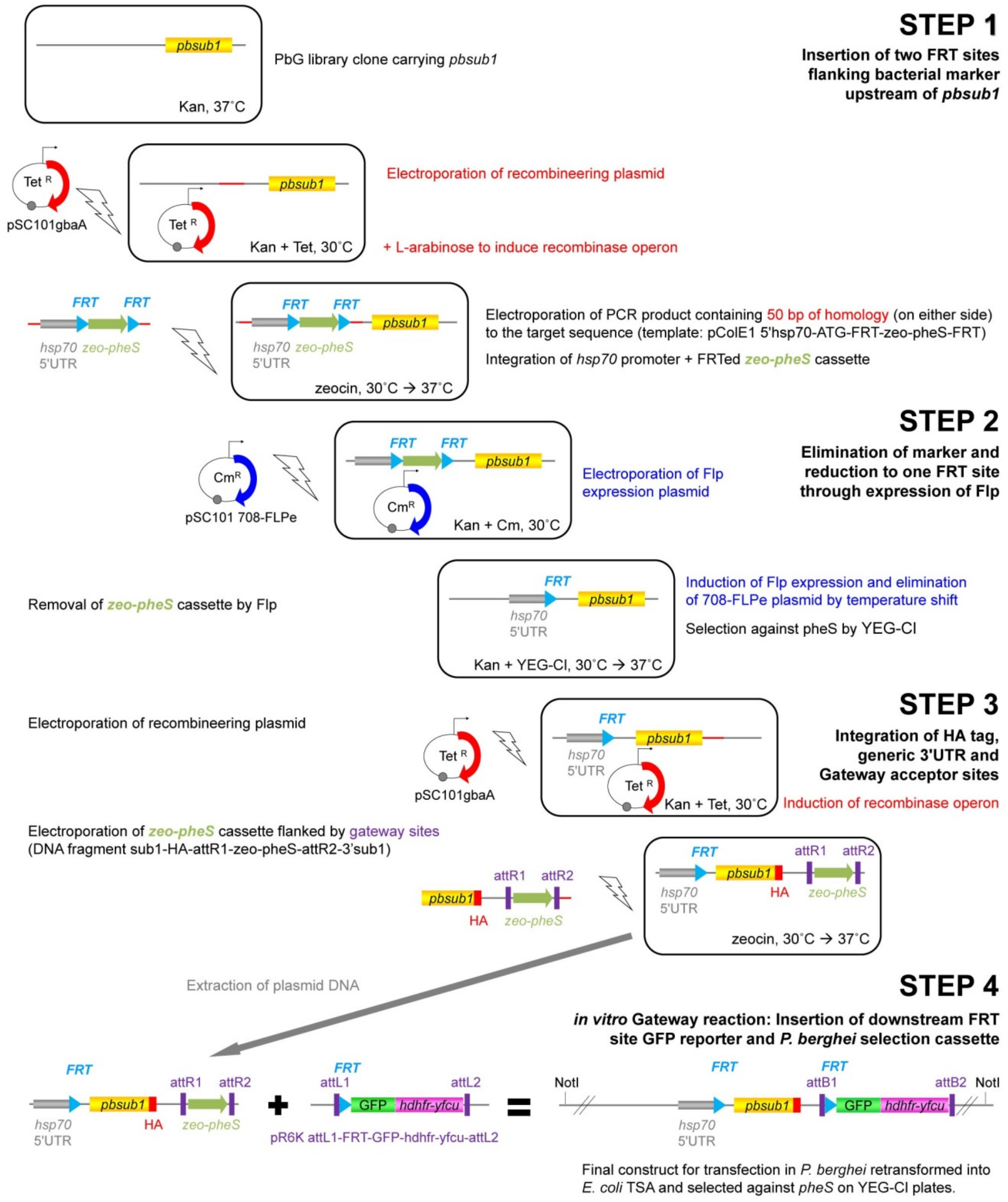

Table S1. Oligonucleotide primers used in this study

Primer name	DNA Sequence (5' to 3')
Vector construction	
F_CatDPbSUB1synth_BamHI	GTGGGTGGATCCTCCATCTACG
R_CatDPbSUB1synth_XhoI	GGCCGCCTCGAGTTATTAATG
F_KI_XhoI	GGGCCCATTTCTTTTTTTACTTTTCCACATGCGAATTCCTTG
F_KI_ApaI	CTCGAGCTGATGAATTAGTAGGAGCTGATTCTATATATGTAG
F_FRT_Zeo_infu_pStep1	ACCAACATTATTATCCCAATAAATTACAATTACAATTAAAAAAAGATGAAGTTCCTATTCTCTA<br>GAAAGTATAGGAACTTCAAGGCGCATAACGATACCA
R_FRT_Zeo_infu_pStep1	AGTAAATAATTCTTCTCCTTTACTGCTAGCTTTTGCGTTAGCCATGAAGTTCCTATACTTTCTAG<br>AGAATAGGAACTTCCCGCCTACTGCGACTATAGAG
F_Zeo_infu_pStep3	TTTCCTTCAATTTCGAAAAGGCGCATAACGATACCAC
R_Zeo_infu_pStep3	CGGCCAGTGAATTCGAAGCTTAATTTGGTAATTTTATAACTTATTAATTTTATAACTTTTAATTT<br>CCCAAACCTTCCGCCTACTGCGACTATAGA
F_pGW_infu1	CGGGCCACCTCGACCTGA
R_pGW_infu1	GAAGTTCCTATACTTTCTAGAGAATAGGAACTTCCCGCGGCAAGCCTGCTTTT
F_pGW_infu2	GAAGTTCCTATTCTCTAGAAAGTATAGGAACTTCATGGCTAGCAAAGGAGAAG
R_pGW_infu2	CCTTCAAACTTGACTTCAGCACGTGT
Recombineering	
F_Step1_rec	GCGTACATCACATTATAATGTGTACATATTGTACAAAATATATAAACCGTGGAAAATATACGTA<br>ATATTTTGTTGGTGA
R_Step1_rec	GATACAAAGAAAATATATGAAAGCAACAAAAGTAATTAACTTGACTATTATTCTTCTCCTTTAC<br>TGCTAGC
F_Step1_short_rec	TATTGTTTTATTTGCCTTTTTTTAATTGTATTTTTTTATATAATGTTAACGGAAAATATACGTAAT<br>ATTTTGTTGGTGA
R_Step1_short_rec	TCAATTTATTATCGTAAATTAAAATGTGAACAATTTTTTCATACAATTTATTCTTCTCCTTTACTG<br>CTAGC
Genotyping PCRs	
Fprom_PbSUB1	GCCCCAAATTTGCCTGTATTGGTC
R_HA	CTTATGCATAATCAGGGACGTCATATGGATAGG
F1_PbSUB1	GGCCTCGAGATGCGAACAGTTTTTATCTATGCTTGTATCATAAG
R1_3'utr	GGCTTTCCTCTTCCGAGTTACTGAG
F2_PbSUB1	GGAGCTCATGTAGAATCTGATGAATTAGTAGG
F_selection	GACATGTTGTTATGCAAGTC
R_ext_1	GCACTTATCAAATTTGATGGATGTAGGTAC
R_ext_2	CCGGCTAGCGAGATAATTCAAATAGATATAACTACCAGAC
R_ext_3	GCTAGCTCAAACTACTAGACGCTTA
F3_PbSUB1	CTGTTCACATCCCCCATCTTCTATTCC
F_KI_XhoI	GGGCCCATTTCTTTTTTTACTTTTCCACATGCGAATTCCTTG
F_KI_ApaI	CTCGAGCTGATGAATTAGTAGGAGCTGATTCTATATATGTAG
F_out_hsp70	GCATTTTACACTATTTTGCCATAAGCACA
R_out GFP	TGTGAGTTATAGTTGTATTCC
R2_out GFP	CCGTATGTTGCATCACCTTC
Southern blot probes	
F_Prom_probe	CCGAAGCTTGCTAGCCCAATGTTCCAAGTGTTTCGTAGCAAAAAGC
R_Prom_probe	GGCAAGCTTGAAAATTCTATTTTTTGTTTGTACGAGAAATAGCTG
F_KI_XhoI	GGGCCCATTTCTTTTTTTACTTTTCCACATGCGAATTCCTTG
F_KI_ApaI	CTCGAGCTGATGAATTAGTAGGAGCTGATTCTATATATGTAG
F_3'utr_pbdhfr_probe	CGTTTTTCTTACTTATATATTTATACCAATTGATTGT
R_3'utr_pbdhfr_probe	TCGAAATTGAAGGAAAAAACATCATTTG
Quantitative RT-PCR	
F_Pb18S	AAGCATTAAATAAAGCGAATACATCCTTAC
R_Pb18S	GGAGATTGGTTTTGACGTTTATGTG
F_HPRT	GTAATGATCGTCAACGGGGGAC
R_HPRT	CCAGCAAGCTTGCAACCTTAACCA

References

1. Withers-Martinez C, Saldanha JW, Ely B, Hackett F, O'Connor T, et al. (2002) Expression of recombinant Plasmodium falciparum subtilisin-like protease-1 in insect cells. Characterization, comparison with the parasite protease, and homology modeling. J Biol Chem 277: 29698-29709.
2. Sajid M, Withers-Martinez C, Blackman MJ (2000) Maturation and specificity of Plasmodium falciparum subtilisin-like protease-1, a malaria merozoite subtilisin-like serine protease. J Biol Chem 275: 631-641.
3. Withers-Martinez C, Suarez C, Fulle S, Kher S, Penzo M, et al. (2012) Plasmodium subtilisin-like protease 1 (SUB1): insights into the active-site structure, specificity and function of a pan-malaria drug target. Int J Parasitol 42: 597-612.
4. Bouillon A, Giganti D, Benedet C, Gorgette O, Petres S, et al. (2013) In silico screening on the 3D-model of the Plasmodium vivax SUB1 protease leads to the validation of a novel anti-parasite compound. J Biol Chem.
5. Janse CJ, Franke-Fayard B, Mair GR, Ramesar J, Thiel C, et al. (2006) High efficiency transfection of Plasmodium berghei facilitates novel selection procedures. Mol Biochem Parasitol 145: 60-70.
6. Blackman MJ, Fujioka H, Stafford WH, Sajid M, Clough B, et al. (1998) A subtilisin-like protein in secretory organelles of Plasmodium falciparum merozoites. J Biol Chem 273: 23398-23409.
7. Amino R, Giovannini D, Thiberge S, Gueirard P, Boisson B, et al. (2008) Host cell traversal is important for progression of the malaria parasite through the dermis to the liver. Cell Host Microbe 3: 88-96.
8. Pfander C, Anar B, Brochet M, Rayner JC, Billker O (2013) Recombination-mediated genetic engineering of Plasmodium berghei DNA. Methods Mol Biol 923: 127-138.
